# Variable fitness effects of bacteriophage resistance mutations in *Escherichia coli:* implications for phage therapy

**DOI:** 10.1128/jvi.01113-24

**Published:** 2024-08-30

**Authors:** Baptiste Gaborieau, Raphaëlle Delattre, Sandrine Adiba, Olivier Clermont, Erick Denamur, Jean-Damien Ricard, Laurent Debarbieux

**Affiliations:** 1 Université Paris Cité, INSERM UMR1137, IAME, Paris, France; 2 Institut Pasteur, Université Paris Cité, CNRS UMR6047, Bacteriophage Bacterium Host, Paris, France; 3 APHP, Hôpital Louis Mourier, DMU ESPRIT, Service de Médecine Intensive Réanimation, Colombes, France; 4 Institut de Biologie de l'ENS (IBENS), École Normale Supérieure CNRS UMR8197, Paris, France; 5 APHP, Hôpital Bichat, Service de Génétique Moléculaire, Paris, France; University of Kentucky College of Medicine, Lexington, Kentucky, USA

**Keywords:** bacteriophage resistance, phage therapy, lung infection, bacterial fitness, mutations

## Abstract

Bacteria exposed to bactericidal treatment, such as antibiotics or bacteriophages (phages), often develop resistance. While phage therapy is proposed as a solution to the antibiotic resistance crisis, the bacterial resistance emerging during phage therapy remains poorly characterized. In this study, we examined a large population of phage-resistant extra-intestinal pathogenic *Escherichia coli* 536 clones that emerged from both *in vitro* (non-limited liquid medium) and *in vivo* (murine pneumonia) conditions. Genome sequencing uncovered a convergent mutational pattern in phage resistance mechanisms under both conditions, particularly targeting two cell-wall components, the K15 capsule and the lipopolysaccharide (LPS). This suggests that their identification *in vivo* could be predicted from *in vitro* assays. Phage-resistant clones exhibited a wide range of fitness according to *in vitro* tests, growth rate, and resistance to amoeba grazing, which could not distinguish between the K15 capsule and LPS mutants. In contrast, K15 capsule mutants retained virulence comparable to the wild-type strain, whereas LPS mutants showed significant attenuation in the murine pneumonia model. Additionally, we observed that resistance to the therapeutic phage through a nonspecific mechanism, such as capsule overproduction, did not systematically lead to co-resistance to other phages that were initially capable or incapable of infecting the wild-type strain. Our findings highlight the importance of incorporating a diverse range of phages in the design of therapeutic cocktails to target potential future phage-resistant clones effectively.

**IMPORTANCE:**

This study isolated more than 50 phage-resistant mutants from both *in vitro* and *in vivo* conditions, exposing an extra-intestinal pathogenic *Escherichia coli* strain to a single virulent phage. The characterization of these clones revealed several key findings: (1) mutations occurring during phage treatment affect the same pathways as those identified *in vitro*; (2) the resistance mechanisms are associated with the modification of two cell-wall components, with one involving receptor deletion (phage-specific mechanism) and the other, less frequent, involving receptor masking (phage-nonspecific mechanism); (3) an *in vivo* virulence assay demonstrated that the absence of the receptor abolishes virulence while masking the receptor preserves it; and (4) clones with a resistance mechanism nonspecific to a particular phage can remain susceptible to other phages. This supports the idea of incorporating diverse phages into therapeutic cocktails designed to collectively target both wild-type and phage-resistant strains, including those with resistance mechanisms nonspecific to a phage.

## INTRODUCTION

Antibiotic resistance poses a significant threat to global public health, heightening the risk of therapeutic challenges and increased mortality for patients worldwide (1). *Enterobacterales*, including the *Escherichia* genus, stand out as a highly concerning multi-drug-resistant (MDR) bacterial family (1). *Escherichia coli* strains have emerged as frequent causative agents of ventilator-assisted pneumonia (VAP) (2, 3). Extra-intestinal pathogenic strains of *E. coli* (ExPEC) implicated in VAP exhibit distinctive traits, such as antimicrobial resistance and virulence factors, and often belong to the ST127/B2 phylogroup (4, 5).

In response to this health challenge, phage therapy—utilizing bacteriophages (phages)—is gaining consideration for treating patients infected with MDR pathogens (6). Extensive experimental models and clinical compassionate-use case series attest to the efficacy of phage therapy (7–12). Notably, in experimental treatments, a single phage dose often matches the efficiency of multiple antibiotic injections (13, 14). However, despite these promising outcomes, the lack of compelling clinical studies hinders wider acceptance (15–17).

Interestingly, the rapid emergence of phage resistance observed in laboratory settings contrasts divergent findings regarding its occurrence (18) or nonexistence (19) in human treatment. It raises inquiries about its relevance in living organisms and concerns about the possibility of phage treatment failure. Successful phage therapy has been demonstrated to rely on a synergistic interaction between phages and the immune system (20). The coordinated assault of phages and immune cells rapidly diminishes bacterial density and reduces the proliferation of phage-resistant clones, which could otherwise lead to the failure of phage treatment when the immune response is compromised (20).

Nevertheless, phage resistance mechanisms could entail trade-off costs, potentially heightening vulnerability to immune cell attacks or inhibiting bacterial growth. In various *in vivo* infection models, instances of phage resistance have been correlated with the attenuation of bacterial virulence (14, 21–24). However, exploring the mechanisms underlying phage resistance and the associated trade-off costs is infrequently undertaken in the context of phage therapy (25, 26).

To unravel the mechanistic insights of phage resistance during phage therapy, we sequenced over 50 phage-resistant clones of *E. coli* strain 536 exposed to virulent phage 536_P1 under both *in vitro* (liquid medium) and *in vivo* (mice lungs) conditions. Our prior findings demonstrated that a single phage dose effectively cured mice with otherwise fatal pneumonia caused by *E. coli* strain 536 (27). Here, we unveil a mutational convergence under both conditions, targeting genes associated with lipopolysaccharide (LPS) (receptor modification, a phage-specific mechanism) or K15 capsule (receptor masking, a phage-nonspecific mechanism) biosynthesis. *In vitro,* fitness examination of phage-resistant clones could not distinguish between the two classes of mutants. In contrast, a virulence assay conducted in mice indicated that LPS-related mutants were nearly avirulent, while K15 capsule-related mutants exhibited virulence levels comparable to the wild-type (WT) strain. Lastly, nonspecific phage-resistant clones display a heterogeneous susceptibility to other phages.

## RESULTS

### A single dose of therapeutic phage selects phage-resistant clones more rapidly *in vitro* than *in vivo*

We sought to assess the occurrence of phage resistance and explore the underlying molecular mechanisms throughout the course of phage treatment. Furthermore, our objective was to distinguish whether the mechanisms through which resistance emerges might differ *in vivo* compared to what we could observe in standard *in vitro* assays. To achieve this, we exposed the ExPEC ST127/B2 phylogroup strain 536 to a single dose of the virulent phage 536_P1 (Myovirus, *Phapecoctavirus*, 149 kb) (27) under two conditions—namely, *in vitro* within a non-limited liquid broth and *in vivo* during a phage therapy treatment for murine pneumonia.

One hundred clones per condition were collected and reisolated from multiple replicates (Table 1). While less than 0.4% of the initial bacterial population exhibited resistance to phage 536_P1, the average resistance rate increased to about 40% after 4 h of incubation in a nutrient-rich liquid medium. In the *in vivo* scenario, 13% of the clones recovered from mice lungs 10 h post-infection (pi) demonstrated resistance. This rate escalated to approximately 40% at 48 h pi (Table 1). Notably, we observed that the bacterial resistance rate displayed heterogeneity among replicates under all conditions, implying the potential involvement of diverse resistance mechanisms (supplementary S1).

**TABLE 1:** Experimental conditions and description of bacterial clones resistant to phage 536_P1^*d*^

Condition	Groups	Time^*a*^ (h)	MOI	Experimental replicates	Bacterial clones per replicate	Bacterial resistance rate (%)	Bacterial clones sequenced	Clones carrying mutation
				*n*	Mean (SD)	Mean (SD)	*n*	*n* (%)
Reference*^b^*		0	-	3	96 (±0)	<0.4 (±0)^*c*^	-	
*In vitro*	536_P1	4	0.1	7	13 (±5)	41 (±30)	18	18 (100%)
*In vivo*	PBS	24	-	8	20 (±0)	<5 (±0)^*c*^	19	0 (0%)
	536_P1	10	3	10	17 (±6)	12 (±16)	17	17 (100%)
	536_P1	48	3	7	20 (±12)	38 (±40)	22	22 (100%)

^*a*^ Time post-infection (phage treatment administered 2 h post-infection).

^*b*^ Reference corresponds to the WT strain 536.

^*c*^ Limit of detection.

^*d*^ MOI, multiplicity of infection; PBS, phosphate-buffered saline; *n*, number; -, not applicable.

Additionally, by collecting clones from the lungs of untreated mice 24 h pi, we obtained a naïve population of bacteria exposed to the mice but not the phage. These clones retained their susceptibility to phage 536_P1, indicating that the immune system selection pressure alone did not influence the emergence of phage resistance (Table 1).

### Genome sequencing reveals a mutational convergence of phage resistance mechanisms toward the modification of two cell-wall components regardless of the environment

After collecting phage-resistant clones, we characterized the molecular mechanisms associated with their resistance. We chose to focus on genomic-based mechanisms, as they will be maintained even after a decline in phage pressure, representing the most potentially problematic contribution to the failure of phage therapy.

The genomes of a subset comprising 57 phage-resistant clones from the abovementioned experiments were sequenced, unveiling a total of 66 mutations, encompassing 53 distinct mutations, each clone carrying at least one mutation (Table 1; supplementary S2). In contrast, all 19 phage-susceptible clones from control mice exhibited no mutations compared to the original WT strain 536 (Table 1). Among the 53 unique mutations, 50 were predicted to impact the function of the encoded proteins, with 40 being truncating mutations and 10 *in silico* predicted as deleterious for protein function. Interestingly, none of these mutations showed a specific association with the origin of samples (*in vitro* vs. *in vivo*) (supplementary S2).

**Fig 1:**
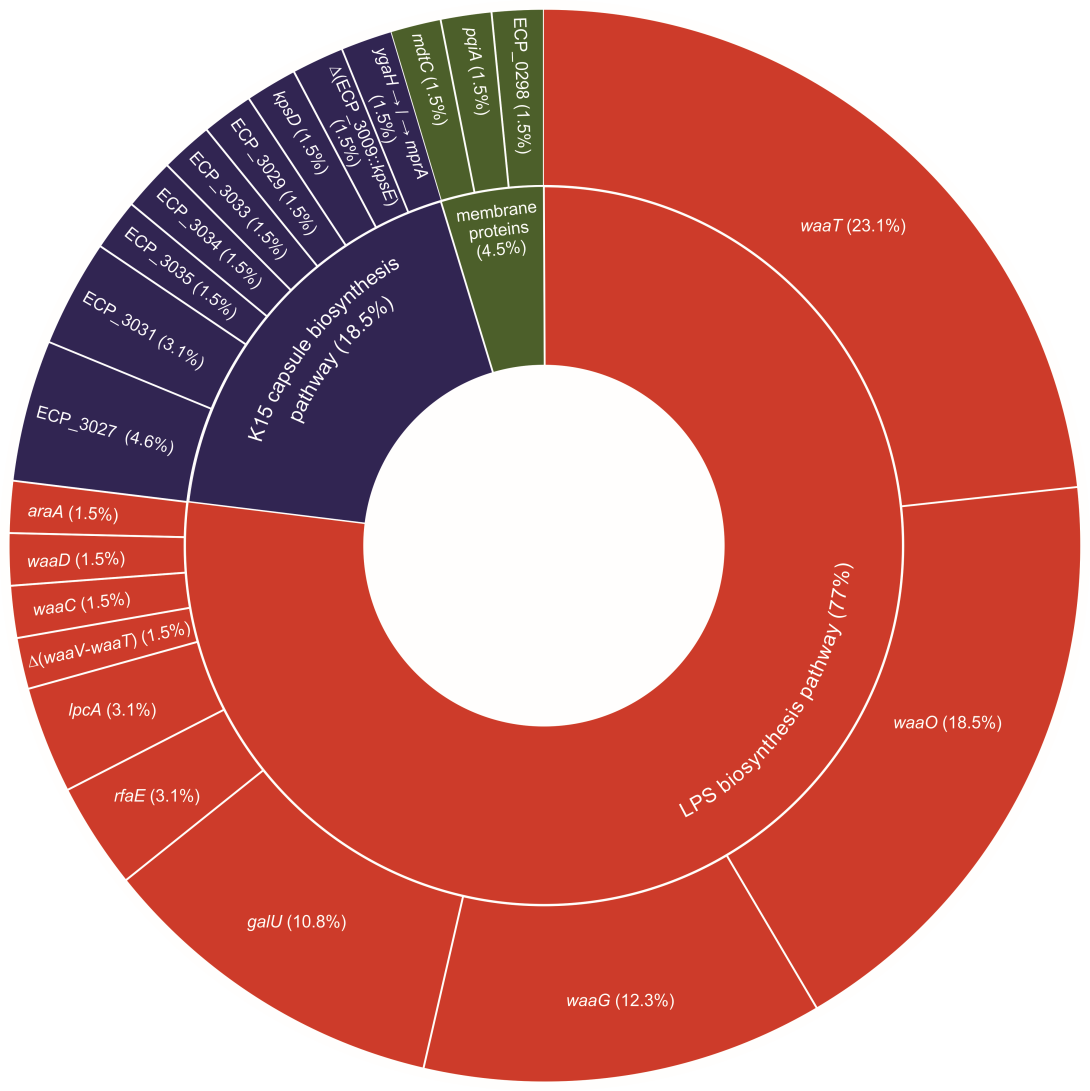
LPS and K15 capsule biosynthesis pathways are the two main targets of mutations identified from phage-resistant clones. The colors represent biosynthetic pathways: red, LPS; blue, capsule; and green, membrane proteins. The percentage of the total number of mutations for each gene is indicated (*n* = 57 clones). LPS, lipopolysaccharide.

A substantial proportion of phage-resistant clones possessed at least one mutation in a gene related to the LPS biosynthesis pathway, constituting 77% of all mutations. The remaining mutations were located in the K15 capsule coding region (18%) and in three other genes (5%) encoding membrane proteins: *mdtC*, which encodes an efflux system protein (28); *pqiA*, involved in a membrane stability system (29); and *ECP_0298*, predicted to be a homolog of a surface adhesin precursor (Fig. 1).

**Fig 2:**
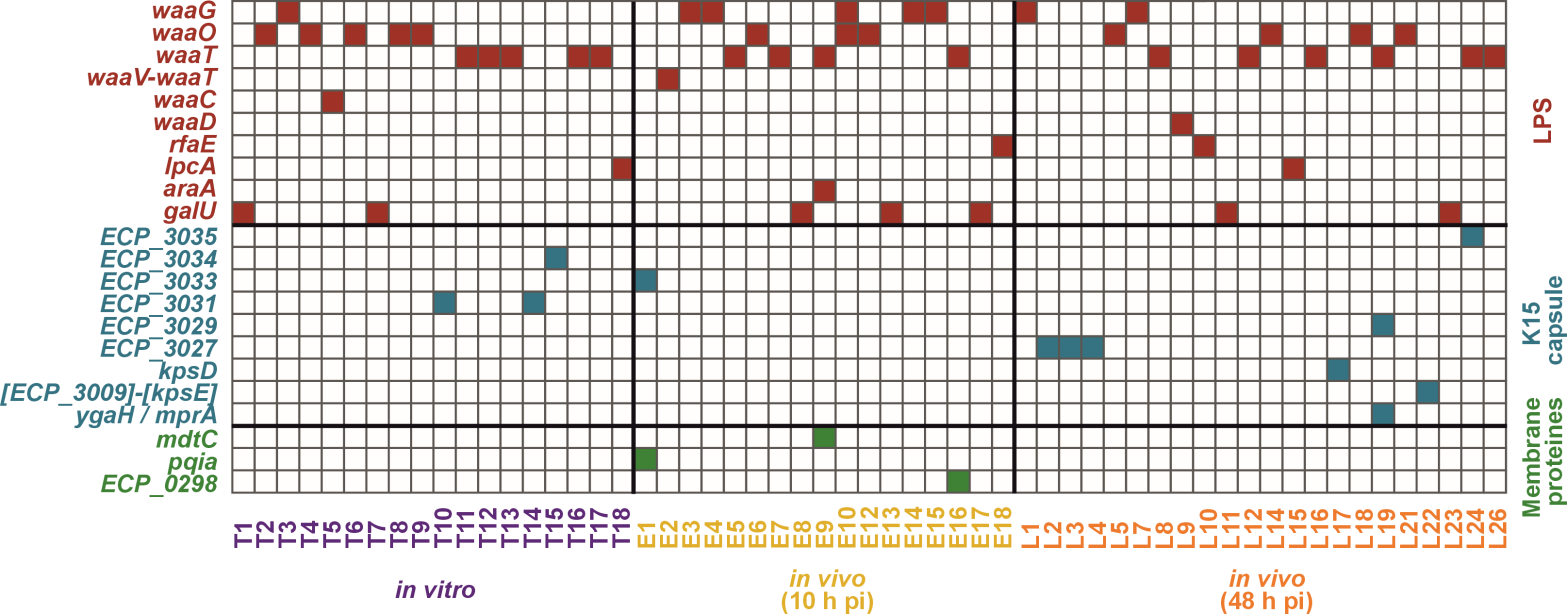
Genome sequencing of the 57 phage-resistant clones reveals a mutational convergence of phage resistance mechanisms at two levels (genes and metabolic pathways). Genes with mutations for each of the 57 clones are grouped based on color-coded conditions (*in vitro*, purple; *in vivo* at 10 h pi, yellow; and *in vivo* at 48 h pi, orange) and color-coded biosynthetic pathways (LPS, red; capsule, blue; and membrane proteins, green). LPS, lipopolysaccharide; pi, post-infection.

Upon plotting the correspondence between mutated genes and the origin of samples (Fig. 2; supplementary S8), it was observed that none of the mutations exhibited a specific association with either *in vitro* or *in vivo* samples. This suggests that phage 536_P1 exerts comparable selection pressure on strain 536, regardless of the environment in which it infects, whether it be a liquid broth or mice lungs. The only notable exception was the presence of three clones mutated in membrane proteins originating from the lungs of infected and treated mice collected at 10 h pi. Nevertheless, these mutations consistently co-occurred with another mutation in a gene involved in LPS or K15 capsule biosynthesis. Despite the involvement of the same pathways (LPS and K15 capsule) under both conditions, the occurrence of strictly identical mutations remained low. Among the 53 different mutations, only three were found under both conditions, indicating a lack of hotspots (supplementary S2). Notably, mutations in LPS or K15 capsule-related genes were predominantly exclusive, with only two out of 57 clones displaying mutations in both pathways (Fig. 2). These findings suggest mutational convergence, irrespective of the condition, indicative of shared selection pressure (30). This also suggests the potential to predict the emergence of phage resistance mechanisms during phage treatment solely from *in vitro* assays.

### Resistance occurs either through a mechanism involving the modification of the phage receptor or through the masking of the receptor mediated by the capsule

LPS, a surface-exposed molecule, plays a pivotal role in phage adsorption, as it serves as a receptor for phages. Consequently, mutations in various genes involved in its biosynthetic pathways are frequently linked to a loss of phage infection (31). The identified mutations are likely to result in varying degrees of truncation of the LPS, as they occur in the *waa* (or *galU*) cluster responsible for assembling the inner and outer cores, or in the *gmH* cluster (*lpcA, rfaE, waaD*) crucial for heptose synthesis (supplementary S3).

**Fig 3:**
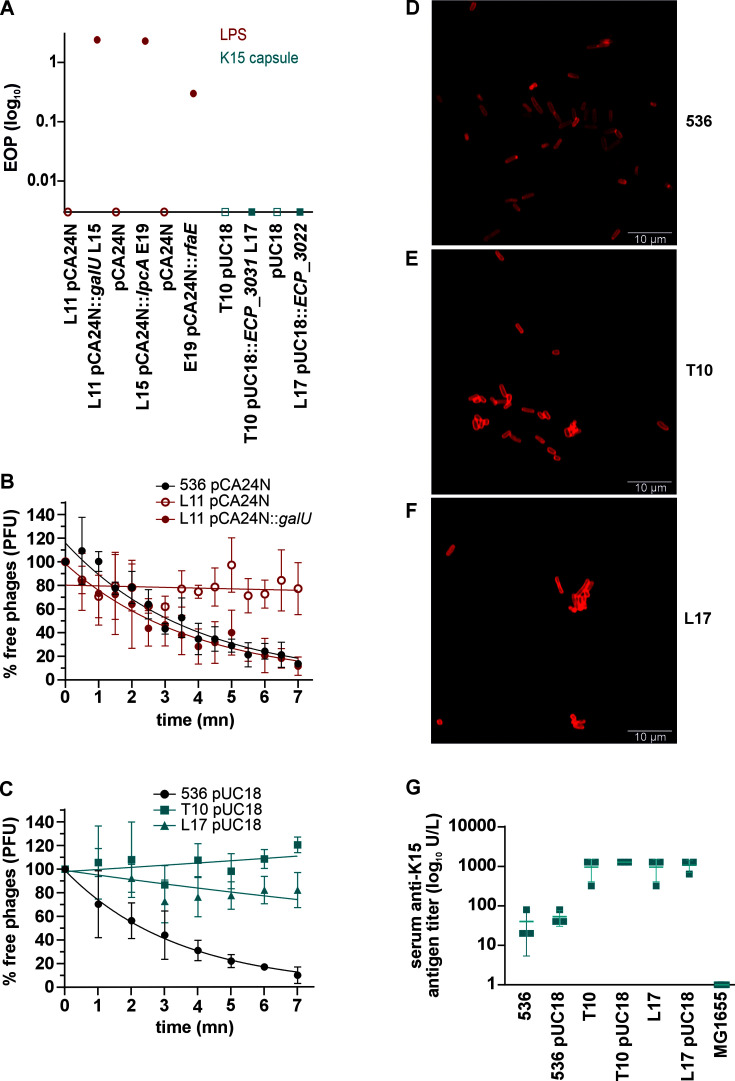
Resistance occurs through either a mechanism involving the modification of the phage receptor (LPS) or an overproduction of the capsule. (**A**) Susceptibility to the phage 536_P1 of representative phage-resistant clones with single mutations in LPS genes (*galU*, clones L11; *lpcA*, clone L15; *rfaE*, clone E19) and in K15 capsule genes (*ECP_3031*, capsular polysaccharide synthesis, clone T10; *ECP_3022*, clone L17). These clones were trans-complemented with the corresponding WT gene cloned in a plasmid (pCA24N or pUC18). Their susceptibility to phage 536_P1 was assayed and compared to WT 536 strains with empty plasmids; the results are expressed as EOP. (**B and C**) Adsorption of the phage 536_P1 on representative clone L11 (*galU*, LPS) and representative clones T10 (*ECP_3031*, K15 capsule) and L17 (*ECP_3022*, K15 capsule). (**D–F**) Detection of K15 capsule expression by immunofluorescence microscopy. The binding of a preabsorbed polyclonal K15 capsule-specific antibody was visualized by an Alexa Fluor 555 Red-labeled secondary antibody and examined by epifluorescence microscopy (phase contrast images, 100×). Compared to the WT 536 strain, clones T10 and L17 have a wider capsule thickness and greater cell agglutination. (**G**) K15 capsule agglutination assays of K15 capsule phage-resistant clones (**L17 and T10**) compared to the WT strain 536 and the non-capsulated strain MG1655. EOP, efficiency of plating; LPS, lipopolysaccharide.

To confirm that phage 536_P1 utilizes LPS as a receptor, we conducted a trans-complementation experiment. We trans-complemented LPS-related mutants, which were fully resistant to the phage [i.e., efficiency of plating (EOP) of 0], with a plasmid expressing the wild-type version of the mutated genes. The susceptibility to phage 536_P1 was successfully restored, showing variable efficiency in plaque formation for all LPS-related mutants (Fig. 3A). The reinstatement of phage adsorption accompanied this restoration of susceptibility (Fig. 3B).

In contrast, a parallel strategy applied to K15 capsule mutants failed to restore phage susceptibility (Fig. 3A) and phage adsorption (Fig. 3C). Capsules play a versatile role in phage-bacteria interactions, as they have been described both as phage receptors and as a layer used to mask these receptors (32, 33). Our observations revealed that only one of the K15 capsule-related mutations affected the operon promoter, while the others influenced polysaccharide synthesis or protein export (supplementary S2). Since we could not demonstrate K15 capsule acting as a receptor for phage 536_P1, we hypothesized that these mutants might enhance capsule production to hinder the recognition of their receptors by phages. Using a K15 capsule-specific serum, we noted that the mutants produced a thicker capsule than the WT strain 536 (see Fig. 3D). Additionally, we observed a pronounced agglutination phenotype by the K15 capsule specific serum in these mutants, differing from the WT strain 536 or a K15-negative control strain (see Fig. 3E through G). These data suggest that the hyperproduction of the K15 capsule in these mutant clones provides a shield against the phage 536_P1.

In summary, we concluded that strain 536 employs two primary strategies to evade phage 536_P1 predation: modifying its receptor or rendering it inaccessible. Given that these two phage defense mechanisms are essentially exclusive, the next step involved assessing the fitness cost associated with these mutations, mainly as they target two *E. coli* virulence factors.

### The fitness cost of phage-resistant clones is dependent on the target of the mutation as well as the environment

To assess the potential fitness cost associated with these various phage-resistant mutations, we examined their behavior in three increasingly complex environments.

**Fig 4:**
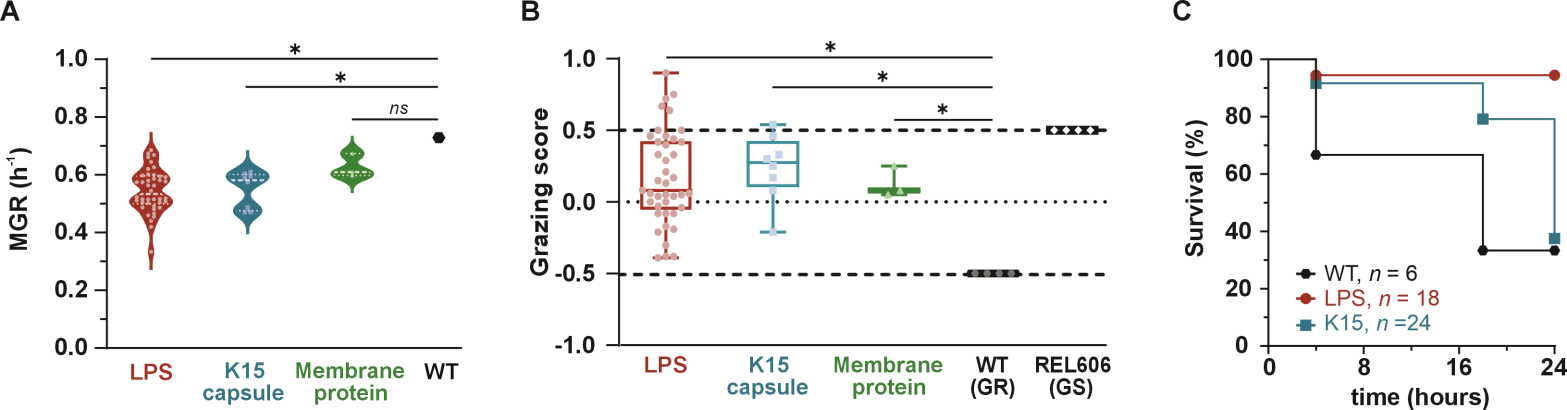
Phage-resistant clones display a broad fitness cost *in vitro* and *in vivo*. (**A**) The maximum growth rate of the 47 clones with a unique set of mutations in a non-limited nutrient medium was calculated from their growth curve recorded every 15 min in a microplate reader for 10 h (*n* = 3 for each clone). (**B**) Protozoan predation by *D. discoideum* of the 47 clones with a unique set of mutations was evaluated by their grazing score (*n* = 3 for each clone). (**C**) Virulence in the acute pneumonia model was assessed toward a subset of clones carrying either single mutations in the LPS (*n* = 3) or the K15 capsule locus (*n* = 3). The survival of mice during 24 h pi (4 × 10^8^ CFU intranasal) by either the WT strain 536 (*n* = 6 mice) or clones carrying single mutations in the LPS biosynthesis pathway (*n* = 6 mice for each clone) or clones carrying single mutations in the K15 capsule locus (*n* = 8 mice for each clone) is represented as Kaplan-Meier. LPS: lipopolysaccharide; MGR: maximum growth rate; GR: grazing resistant; GS: grazing susceptibility; pi, post-infection.

Firstly, we measured the maximum growth rate (MGR) in a non-limited nutrient medium for all clones with a unique set of mutations (*n* = 47). Compared to the WT strain 536, which has an MGR of 0.73 h⁻¹, clones mutated in LPS or K15 capsule biosynthetic pathways displayed significantly lower MGR, with a median of 0.53 h⁻¹ (range 0.33–0.68 h⁻¹; *p* = 0.027) and 0.58 h⁻¹ (range 0.47–0.61 h⁻¹; *p* = 0.042), respectively. Meanwhile, the three clones mutated in membrane proteins exhibited a median MGR of 0.61 h⁻¹ (range 0.60–0.67 h⁻¹; *p* = 0.349), similar to the WT strain 536 (Fig. 4A; supplementary S4). To verify that the observed difference in MGR is not due to a bias in the measurement technique (absorbance), we ensured that the correlation between the measured optical density (OD) and the amount of colony-forming units (CFU) did not vary under the three different bacterial states (WT, LPS mutation, and capsule mutation). This possibility was discarded when absorbance-based inoculums were plated to test virulence in the two other settings (see below).

Secondly, we utilized *Dictyostelium discoideum*, a unicellular organism mimicking macrophage phagocytosis (34–36), to assess the grazing resistance (GR) phenotype of the clones mentioned above (*n* = 47). The WT strain 536 is fully resistant to amoeba phagocytosis, exhibiting no lysis plaques (GR phenotype) at densities of 10², 10³, 10⁴, and 10⁵ amoebae per 10⁸ CFU of bacteria. In contrast, the control strain REL606 is susceptible to grazing (GS phenotype), characterized by large lysis plaques and the formation of fruit bodies (supplementary S5). All the mutated clones showed increased susceptibility to *D. discoideum* compared to the WT strain 536, with some being even more susceptible than the GS control. However, no significant difference in the amplitude of the grazing phenotype was observed between LPS and K15 capsule mutants (*p* = 0.80) (Fig. 4B).

Finally, we investigated whether the virulence of phage-resistant clones toward our murine pneumonia model could be affected. Six mutated clones carrying single mutations either in the LPS (*galU*, clone T1; *waaG* clone E15; *waaT*, clone T11) or in the K15 capsule [*ECP_3033*, clone E1; *ECP_3027*, clone L3; Δ(*ECP_3009–kpsE*), clone L22] were selected. Kaplan-Meier survival curves of mice infected by these clones revealed, compared to the WT strain 536 (*n* = 6 mice; 33% survival at 24 h pi), a significant decrease in the virulence of the LPS mutated group of clones (*n* = 18 mice; 94% survival at 24 h pi, *p* = 0.001), while the K15 capsule mutated group of clones remained virulent (*n* = 24 mice; 62.5% survival at 24 h pi, *p* = 0.24) (Fig. 4C). Results remain similar when plotted for each mutated gene (supplementary S6a). As expected from the survival curves, the mean level of CFU (±SD) in mice lungs at 24 h pi is 3-log lower for LPS mutants (5.25 ± 1.39) compared to both WT strain 536 (8.73 ± 1.23) and K15 capsule mutants (8.57 ± 2.36) (supplementary S6b). Therefore, while the above two *in vitro* assays do not discriminate between the two classes of mutants, the *in vivo* virulence assay clearly segregates LPS from K15 capsule mutants. This suggests that anticipating virulence trade-off costs cannot be easily achieved through *in vitro* assays alone.

### Unspecific phage resistance mechanism emerging from a single phage does not provide a broad resistance to multiple phages

**Fig 5:**
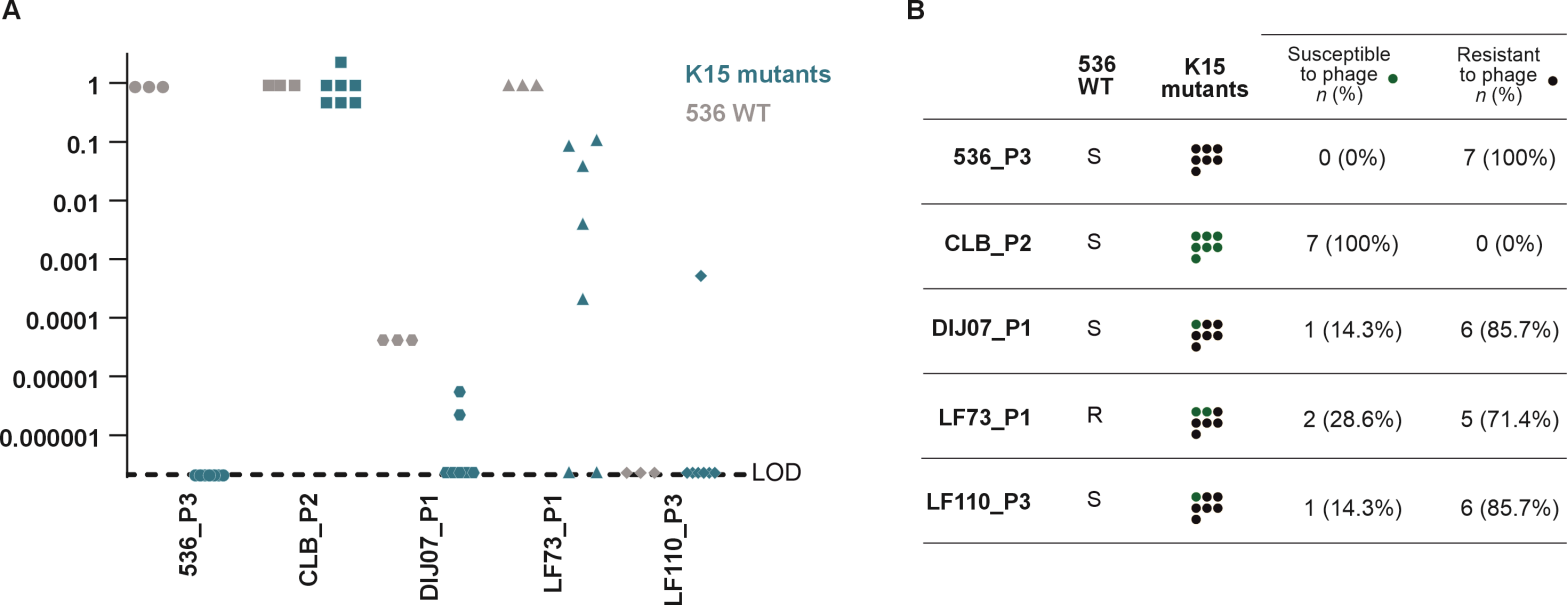
Phage 536_P1 K15 capsule-resistant clones display uneven susceptibility to five other phages. (**A**) Efficiency of plating (EOP) of five virulent phages (536_P3, CLB_P2, DIJ07_P1, LF73_P1, and LF110_P3) on seven phage 536_P1-resistant clones carrying a mutation in K15 capsule biosynthetic genes and three 536 WT clones. The EOP values correspond to the ratio of phage titers on the tested clone over the phage host strain. LOD, limit of detection of 10^−7^. (**B**) Synthetic representation of the EOP values from (**A**) with a cut-off value of 0.1 to differentiate susceptible (green) to resistant (black) phenotypes.

The selection of K15 capsule phage-resistant clones that retain a virulence comparable to the WT strain 536 raises concerns about the potential failure of phage treatment. To address this, we investigated whether other phages could effectively infect these clones as a countermeasure. Five virulent phages (536_P3, CLB_P2, DIJ07_P1, LF73_P1, and LF110_P3) (supplementary S7), previously isolated and characterized in our laboratory for their ability to form plaques on a broad collection of *E. coli* VAP strains (4), and which safety was evaluated (37), were selected for this analysis. Four can lyse the wild-type strain 536 among these five phages, whereas one phage (LF110_P3) cannot infect the original WT strain 536 (Fig. 5).

We evaluated the plaque-forming ability of these five phages on the seven 536_P1-resistant clones, each harboring distinct mutations targeting K15 capsule-related genes (Fig. 5A). The phage susceptibility coverage ranged from 0% (536_P3) to 100% (CLB_P2). While the susceptibility of K15 capsule 536_P1-resistant clones remained intact for certain phages (e.g., CLB_P2, EOP around 1), it slightly decreased for others (LF73_P1 and DIJ07_P1, showing a 1- to 2-log decrease in EOP) and completely abolished for 536_P3 (Fig. 5B). This suggests that overproducing the K15 capsule does not offer broad and robust protection against many phages. Intriguingly, LF110_P3 infected one K15 capsule-related mutant, albeit with low efficacy (Fig. 5).

Moreover, similar findings have been observed when testing LPS mutants (supplementary S9). Consequently, when strain 536 responds to phage 536_P1 exposure by modifying LPS or capsule, these phage resistance mechanisms could be easily circumvented using additional phages, even if they are not specific to a given phage.

## DISCUSSION

Phage therapy holds promise for aiding patients infected by MDR bacteria (6). Nevertheless, the current application of phages is somewhat empirical due to the absence of conclusive clinical trials. Additionally, monitoring phage resistance and related mechanisms in patients undergoing treatment is often overlooked despite the potential critical impact on the success of phage therapy (25, 26). In this study, we examined a large population of phage-resistant clones from both *in vitro* and *in vivo* conditions, assessing their mutational fitness costs. Phage resistance not only consistently leads to the selection of less virulent clones but, contrary to some reports (14, 23, 24, 38, 39), also favors the emergence of clones maintaining virulence comparable to the wild-type strain. This highlights the need for systematic monitoring of this phenomenon during treatment.

Sequence analysis of phage-resistant clones revealed a marked convergence toward only two biosynthetic pathways—LPS or K15 capsule. However, the scarcity of identical mutations suggests a lack of hotspots, indicating independent selection events in each experiment. While these pathways are known targets bacteria use to defend against phages (14, 21–24, 39–41), our findings show that LPS and K15 capsule mutants employ distinct mechanisms to confer phage resistance. LPS mutations likely disrupt phage receptor synthesis, while K15 capsule mutants overproduce capsule synthesis, potentially masking the receptor. Given that approximately half of bacterial genomes encode at least one capsule biosynthetic cluster (42), especially in extra-intestinal virulent B2 phylogroup *E. coli* strains (43), the overproduction of capsules for phage resistance might be more common than previously thought. It is plausible that the limited number of analyzed phage-resistant clones in previous studies might have hindered the identification of less frequent phage-resistant mutants (26). Our observations could also be specific to the particular phage/bacteria pair studied, and we cannot exclude this possibility.

It is anticipated that phage resistance mechanisms affecting the bacterial envelope could impose a fitness cost compared to the wild-type phage-susceptible strain (44–47). This was observed in all mutants when measuring growth rates or grazing resistance to amoeba. Surprisingly, there were no significant fitness differences between LPS and K15 capsule mutants concerning amoeba grazing. Despite LPS and capsule being envelope components contributing to the pathogenicity of strain 536 (48–50), there was an initial expectation that LPS mutants would experience a more pronounced impact than capsule mutants. Even though serum resistance is directly tied to the presence of the capsule in strain 536 (51) and contributes to grazing resistance (36), a previous study indicated that mutants of strain 536, carrying increasing deletions of pathogenicity islands, did not show reduced resistance to grazing (52). This suggests that the grazing test might not be sensitive enough compared to the *in vivo* virulence assay. The virulence assay revealed that capsule mutants maintained virulence levels comparable to the wild-type strain, whereas LPS mutants became avirulent. Consequently, the selection imposed by phage 536_P1 on strain 536 results in two populations of resistant clones exhibiting divergent behaviors toward the animal host. However, due to the lower frequency of capsule mutants compared to LPS mutants, the overall success of the phage treatment is preserved (27).

The time required to reach 40% of mutants *in vivo* (48 h) was much longer than *in vitro* (4 h), primarily due to the time necessary for the phage to reach and infect a substantial population of susceptible bacteria, along with the duration needed for phage-resistant clones to proliferate. Nonetheless, under both studied conditions, the selection of phage-resistant clones resulted in mutations affecting the same biosynthetic pathways. We also noted considerable variation in the proportion of phage-resistant clones (ranging from 0% to 100%) isolated at 48 h from different animals, suggesting that the host could influence the selection process. Similar variability has been observed in murine models of different infections, such as sepsis by *Acinetobacter* or *Klebsiella* (53–55). Thus, while the bacterial targets appear consistent for a phage-bacteria pair, the emergence of phage resistance during infection may be influenced by host/pathogen/phage dynamics.

Our acute pneumonia model has limitations, including using a large bacterial population to initiate a fatal infection by overwhelming the immune system and administering a large phage dose for rapid and efficient treatment (56). It remains possible that a potential impact of the animal host on phage resistance mechanisms could not be detected within this setting and may necessitate extended observation time. Nevertheless, we observed that the frequency of K15 capsule mutants was higher at 48 h compared to 10 h pi, aligning with their unaffected virulence. In an immunodeficient context, the growth of capsule mutants could potentially lead to treatment failure. Ultimately, the limited data found in the literature regarding instances in humans where treatment solely with phages fails may contribute to the absence of identified cases wherein phage resistance emerges without affecting bacterial virulence.

Overall, the presented data suggest that characterizing phage-resistant mutants selected *in vitro* could be adequate to identify, at least, the primary mechanisms by which bacteria evade phage predation during the initial phase of treatment, aligning with recent compassionate treatment approaches (47, 57). While LPS-related mutations tend to occur more frequently than other target mutations with associated fitness costs *in vitro*, their prevalence diminishes in competitive environments, as evidenced by comparisons of mutations from fluctuation and evolution experiments in *E. coli* (45). In contexts like phage therapy, the success of mutations conferring resistance to phages could significantly be influenced by their pleiotropic effects on bacterial virulence, thereby complicating the predictability of therapeutic outcomes.

A proposed strategy to counteract the selection of phage-resistant mutants involves utilizing multiple phages, commonly known as phage cocktails. However, the criteria for selecting phages in a cocktail vary depending on the application. Presently, compassionate treatments often incorporate several phages chosen for their ability to infect the patient's specific strain. At the same time, ready-to-use cocktails available in Georgia comprise phages targeting different bacterial species (58). Our study revealed that some phage-resistant clones become susceptible to phages that cannot infect the WT strain 536, a phenomenon recently reported for *Acinetobacter* strains when resisting a first phage (46, 53). These findings suggest that phage cocktails may not necessarily have to include only phages targeting the patient's specific strain. Considering phage therapy within the broader context of phage-bacteria coevolution opens up opportunities for each phage in a cocktail to potentially become the most efficient at some point (59).

In conclusion, we foresee that conducting prompt analyses of phage-resistant mutants emerging *in vitro* is crucial for each therapeutic phage. This process should aid in developing robust phage cocktails intended for clinical use.

### Research in context

#### Evidence before this study

Before this study, in February 2023, we conducted a comprehensive literature search (Medline and Web of Science) on preclinical and clinical studies addressing the emergence of phage resistance during phage therapy. Our search utilized terms such as “bacteriophage,” “therapy,” and “resistance”. Among these studies, only a limited number delved into the characterization of resistance mechanisms, even in cases where phage treatment failed.

In instances of compassionate use in humans, bacterial resistance to phages often manifests through modifications to the bacterial surface, hindering phage-bacteria interactions. Notably, successful treatment outcomes were observed when the fitness cost of the resistant clones played a role (12). Preclinical models focusing on various infections (sepsis, pneumonia, wound, meningitis, or endocarditis) or targeting intestinal colonization consistently indicated that phage resistance correlated with alterations to the surface receptor and incurred a fitness cost (either in virulence or growth). Some studies (39, 57) suggested similarities between phage resistance mechanisms observed during treatment and those identified in *in vitro* co-culture setups. Unfortunately, most studies focused on a limited number of isolated resistant clones, resulting in restricted data on the emergence of phage-resistant clones during treatments. Estimations of frequency, emergence rate, and diversity of resistance mechanisms in environments involving tripartite interactions between bacteriophages, bacteria, and hosts were notably sparse.

#### Added value of this study

This study isolated more than 50 phage-resistant mutants from both *in vitro* and *in vivo* conditions, exposing an extra-intestinal pathogenic *E. coli* strain to a single virulent phage. The characterization of these clones revealed several key findings: (1) mutations occurring during *in vivo* phage treatment affect the same pathways as those identified in *in vitro* assays (2); the resistance mechanisms are largely associated with modification of two cell-wall components, with one involving receptor deletion (phage-specific mechanism) and the other, less frequent, involving receptor masking (phage-nonspecific mechanism) (3); *in vitro* fitness phenotypes were unable to distinguish between the two resistance mechanisms. However, an *in vivo* virulence assay demonstrated that the absence of the receptor abolishes virulence while masking the receptor preserves it (4); and clones with a resistance mechanism nonspecific to a particular phage can remain susceptible to other phages. This supports the idea of incorporating diverse phages into a therapeutic phage cocktail designed to collectively target both wild-type and phage-resistant strains, including those with resistance mechanisms nonspecific to a phage.

#### Implications of all the available evidence

The study recognized that specific resistance mechanisms to phages, albeit a minority, pose a potential risk for failure during phage therapy. Consequently, we propose that the phage cocktail design should incorporate phages that are effective against potential future phage-resistant clones. This strategic inclusion aims to mitigate or prevent the growth of these resistant clones.

## MATERIALS AND METHODS

### Bacterial strains, phages, and culture conditions

The *E. coli* strain 536 (4,938,920 bp; NC_008253.1) classified within the B2 phylogenetic group possesses the O6:K15:H31 serotype and corresponds to sequence type 127 (60). The phage 536_P1 (149.4 Kb; OZ035728.1) (27) was amplified using the host strain 536 and purified through adapted molecular biology protocols, including a step of endotoxin removal using EndoTrap (Lionex, Germany). The resulting stock solution, with a concentration of 3.1 × 10^10^ PFU/mL (endotoxin concentration of 2.15 EU/mL), was then diluted in phosphate-buffered saline (PBS).

Phages CLB_P2 (OZ035781.1) (61), LF73_P1 (OZ035729.1), LF110_P3 (OZ035766.1), and DIJ07_P1 (OZ035743.1) belong to the *Guelin* collection (62). Phage 536_P3 is an additional phage isolated on the same host strain 536 than the phage 536_P1.

Unless specified otherwise, bacterial strains were cultivated at 37°C on either liquid or semi-solid agar Lennox Broth medium (LB) (Becton Dickinson, USA). Drigalski agar medium (Bio-Rad, France) was utilized for bacterial selection and counting from mice lungs. For amoeba experiments, bacteria were cultured in a glucose-HL5 medium (Formedium, UK).

### Isolation of phage-resistant clones

#### From *in vitro* condition

A freshly prepared exponential bacterial culture from a single *E. coli* strain 536 clone (*n* = 7), obtained from a glycerol stock, was cultured in LB medium under constant agitation at 37°C. Subsequently, 5 × 10^7^ PFU of bacteriophage 536_P1 was added to the 10 mL bacterial culture containing 5 × 10^8^ CFU. The growth of the bacterial culture was then tracked by measuring turbidity using a spectrophotometer (Novaspec II, Pharmacia LKB, UK) at regular intervals. Upon reaching the nadir, the remaining bacterial cells were centrifugated (4,000 g, 15 min, 4°C), and the supernatant was removed. The resuspended pellets were then plated on LB agar plates and incubated overnight at 37°C.

#### From *in vivo* condition

We employed a previously published murine model for pulmonary phage therapy (27). Mice, anesthetized with a combination of ketamine and xylazine through intraperitoneal administration, were infected by intranasal inoculation with 1 × 10^8^ CFU of *E. coli* strain 536 (0 h). Two hours later, mice, anesthetized with 2% isoflurane inhalation, were either subjected to intranasal administration of 20 µL of 536_P1 (equivalent to 3 × 10^8^ PFU) in the treated group (*n* = 17) or received an intranasal administration of PBS in the control group (*n* = 8). The number of mice in each group was determined to achieve a reduction in mortality from 70% to 0% at 24 h pi compared to the untreated group, with an alpha risk of 5% and a power of 80%. This required a minimum of six mice per group. Mice were hydrated subcutaneously with 150 µL of 0.9% NaCl (Aguettant, France) and monitored twice daily for weight loss and behavior. The treated group was divided into an early follow-up group (10 h pi, *n* = 10) and a late follow-up group (48 h pi, *n* = 7). For the control group, mice were sacrificed at 24 h pi by intraperitoneal injection of 150 µL of sodium pentobarbital (Ceva Santé Animale, France). Mice were housed in groups of three to five individuals per cage. Allocation to different groups was determined randomly based on the number of cages after a 1-week acclimatization period. Lungs were aseptically recovered, weighed, and homogenized in PBS 1× using a FastPrep 25-5G (MP Biomedical, USA). The homogenates were centrifuged (4,000 g, 15 min, 4°C), and the resuspended pellets in PBS 1× were plated on Drigalski agar plates and incubated at 37°C.

### Determination of bacterial susceptibility to phage

For each animal and *in vitro* experiment, a maximum of 20 clones were randomly selected and re-isolated three times on LB agar. The susceptibility to 536_P1 was assessed in both liquid and solid media.

#### Bacterial growth kinetics in liquid medium

Bacterial growth was monitored by recording OD_600 nm_ readings every 15 min at 37°C under agitation for 10 h using a microplate spectrophotometer (Tecan Infinite F200 pro, Switzerland). The OD_600 nm_ for each clone under two conditions (with and without phage) was measured in three different wells. The starting conditions were standardized: from a saturated culture, the bacterial culture was refreshed to achieve exponential growth, and the OD_600 nm_ was adjusted to 0.1. Subsequently, 150 µL was taken to fill a 96-well plate (equivalent to 4.5 × 10^6^ CFU). Ten microliters of bacteriophage (1.5 × 10^6^ PFU) were added to experimental wells, and 10 µL of PBS 1× were added to control wells. A positive control (WT *E. coli* strain 536) was included in each microplate. The clones were categorized into three groups based on their growth curve profiles: susceptible (equivalent to the growth curve of the WT *E. coli* strain 536 infected by 536_P1), resistant (equivalent to the growth curve of WT *E. coli* strain 536 not infected), and intermediate (any profile different from the two above).

#### Efficiency of plating (EOP) in solid medium

Bacteriophage 536_P1 was serially diluted (10-fold) in PBS 1×, and 4 µL drops of each dilution were spotted on LB agar plates previously covered by a bacterial lawn of either the WT strain 536 or each mutant individually. The EOP was calculated as the ratio of the titer on a given mutant over the titer of the WT strain. Three replicate experiments were conducted. A ratio of less than 0.1 indicates the presence of a resistant phenotype, as defined previously (63).

### DNA extraction and whole genome sequencing

Bacterial DNA extraction was performed using the EZ1 DNA tissue kit (Qiagen, Germany) on the EZ1 Advanced XL system (Qiagen, Germany), following the manufacturer's guidelines. The libraries were prepared using the Nextera XT DNA Library Preparation Kit (Illumina, USA) per the previously outlined protocol (64). Subsequently, pair-end sequencing with a fragment size of 300 bp was carried out on the MiSeq platform (Illumina, USA).

### Bioinformatics analysis of mutants

Reads quality was assessed using FastQC (v0.11.8) (65), followed by assembly with SPAdes (v3.11.1) (66). *In silico* typing of the clones was performed using SRST2 (v0.2.0) (67), confirming the sequence type (ST) according to MultiLocus Sequence Typing (MLST) Pasteur (ST33) and Warwick (ST127), as well as the serotype (O6H31). The Clermont Typing software (68) confirmed phylogroup B2 for all phage-resistant clones.

This study utilized the WT strain 536, which was sequenced alongside phage-resistant clones. Comparisons to the referenced genome (NC_008253.1) revealed mutations in the WT strain, which were integrated into the reference genome using Breseq's gdtools (v0.33). Mutations were identified by comparing reads for each phage-resistant clone to the updated reference genome using Breseq Variant Report (v0.33) (69).

Each single nucleotide polymorphism (SNP) detected was manually reviewed. A search for protein-to-protein interactions using the STRING database (70) was conducted for mutations located in genes of unknown function. The functional consequence of mutations was predicted using SIFT (v1.3) (71), PolyPhen-2 (v2.2.2) (72), and PROVEAN (v1.1.3) (73). A mutation was considered potentially harmful if at least two predictions were concordant (EIPD score < 0.05, a PolyPhen-2 result of “probably damaging” or “potentially damaging,” and a PROVEAN score ≤ 2.5).

### Complementation of LPS and K15 capsule mutants

A subset of phage-resistant clones carrying a single mutation in a gene associated with the LPS biosynthetic pathway (*galU*, L11; *lpcA*, L15; *rfaE*, E19) were complemented using the corresponding ASKA plasmids (pCA24N backbone, chloramphenicol 25 µg/mL) (74). To complement K15 capsule-related phage-resistant clones (*ECP_3022*, L17; *ECP_3031*, T10), the corresponding open reading frames (ORFs) were amplified from the WT strain 536 and cloned into the pUC18 plasmid (ampicillin 100 µg/mL) using *BamHI* and *SphI* enzymes (New England Biolabs, USA) (ECP_3022: F-GGATCCGAATGAGTTTGTGATGAAATTA, R-GCATGCTTACAAAGACAGAATCACTTTT; ECP_3031:

F-GCATGCTTAAATTTCTGAGTACGGCAAA, R-GGATCCAATGGTGAAATATGAAAATCAA). The cloned open reading frames (ORFs) were sequenced, and the resulting plasmids were then transformed into the corresponding mutants.

### Phage adsorption

The adsorption of phage 536_P1 was evaluated following a previously described method (75). In summary, cells were infected at a low multiplicity of infection (MOI) of 0.1 and incubated for 10 min at 37°C with constant agitation. Samples of 50 µL were collected every 30 s until 7 min and immediately placed on ice before titration on the WT strain 536 to determine the amount of free phages over time.

### K15 capsule staining

Overnight bacterial cultures were resuspended in 1 mL of PBST (PBS 1× with 0.1% Tween 20 detergent), followed by centrifugation at 8,000 g for 2 min at room temperature to remove excess media. The resulting pellet was then resuspended in 500 µL of PBST, from which 10 µL was used to obtain a thin smear on a glass slide that was air dried for 30 min. Lysozyme solution (10 mg/mL) was applied onto the slide and incubated at 37°C for 15 min. Next, blocking was performed using 5% BSA in PBST for 1 h at room temperature. The primary antibody (Ab1 K15, Product 55350, SSI Diagnostica, Denmark) was diluted 1/500 in PBST and allowed to bind for 1 h at room temperature. After three washes with PBST, the secondary antibody [goat anti-rabbit IgG (H + L) Alexa Fluor Plus 555 (ThermoFischer Scientific, USA)] was applied at a dilution of 1/200 for 1 h at room temperature followed by three washes with PBST. Slides were mounted using ProLong Gold mounting medium (Life Technologies, USA) and images were collected with inverted microscope (Olympus IX81) equipped with a 100× objective lens.

### K15 antiserum agglutination assay

Bacterial agglutination assay using a K:15 antiserum (Product 55350, SSI Diagnostica, Denmark) was conducted following a previously described procedure, with determination of the cut-off titer for the K:15 antiserum (76). In brief, 5 µL of twofold serial dilutions of the K:15 antiserum (ranging from 1:20 to 1:2,560) was mixed with 10 µL (1.10^7^ CFU) of each tested bacterium. The mixture was incubated at 37°C without agitation for 1 h. followed by overnight incubation at 4°C. After overnight incubation at 4°C, agglutination was observed, with the highest serum dilution yielding positive detection.

### Fitness assays

#### Growth rate in non-limited liquid medium

For each strain, a single colony was selected from an agar plate to inoculate a single well of a 96-well plate filled with 150 µL of LB and incubated overnight under agitation at 37°C. Subsequently, 15 µL from each well was transferred into a new plate filled with 150 µL of LB and introduced into a microplate spectrophotometer to record OD_600 nm_ every 15 min at 37°C under regular agitation for 10 h. Three independent replicates were performed for each clone.

The growth curves were analyzed using R software. The initial time point was normalized for each curve as the first OD_600 nm_ measurement was set to 0.1. The time series was then smoothed using the *smooth.spline* function, and the first and second derivatives were calculated with respect to time, expressed logarithmically. All time points at which the second derivatives changed sign (indicating local maximum or minimum growth rates) were identified. These points correspond to the maximum growth rate (MGR) expressed in h^−1^. Smoothing was employed to reduce the impact of measurement noise on the maximum growth rate.

#### Dictyocellium discoideum grazing assay

The amoeba *D. discoideum* axenic strain AX3 and associated methods were previously detailed (36). Fresh bacterial exponential cultures, cultivated in 10 mL glucose-HL5 medium at 37°C under agitation, were washed in 10 mL of MCPB buffer and adjusted to a bacterial concentration of 1 × 10^8^ CFU in 300 µL. This bacterial suspension was plated on glucose-HL5 medium agar (55 mm diameter dishes). A fresh 24 h culture of *D. discoideum* cells, grown in 10 mL of glucose-HL5 medium at 23°C with no agitation, was washed with 10 mL of MCPB buffer and adjusted to five concentrations corresponding to 10, 10^2^, 10^3^, 10^4^, and 10^5^ amoebae in 300 µL. These amoebae concentrations were overlaid on the agar plates previously covered by bacterial lawns. The plates were then incubated at 23°C and examined at 3 and 6 days to observe the appearance of lysis plaques, indicating the phagocytosis of bacteria by the amoeba and defining the grazing susceptibility (GS) phenotype. Strain *E. coli* REL606 was used as a positive control. The lack of phagocytosis defines the grazing-resistant (GR) phenotype, considering that the WT strain 536 was previously reported as GR. Results were expressed as a grazing score (number of conditions with the appearance of lysis plaques: 10^2^, 10^3^, 10^4^, 10^5^, and sporulation at day 6). The scores were normalized for each independent experiment from −0.5 (grazing score of 536 WT) to 0.5 (grazing score of REL 606). Three independent replicates were performed for each strain.

#### Virulence assay in mice

BALB/cJRj mice (*n* = 48) were infected following the previously described procedure and were monitored for weight loss and behavior three times within 24 h. After this monitoring period, the mice were sacrificed to collect lungs, which were processed as described earlier to count bacteria. Two distinct replicates of 24 mice each were conducted. The determination of the number of mice in each group aimed to achieve a reduction in mortality from 70% to 0% at 24 h between the untreated group and each of the treated groups. This was done with an alpha risk of 5% and a power of 80%, requiring a minimum of six mice per group.

### Statistical analysis

The results are presented as the median and range, mean and standard deviation, or the number and percentage of individuals. No pre-established exclusion criteria were applied, and all sample and data points were included in the analysis.

Multiple correspondence analysis (MCA) was conducted using the *mca* function of the FactoMineR package (v1.34) (77). The MCA generated a simultaneous display on a two-dimensional representation of 57 observations (clones with at least one mutation) and 25 qualitative variables (mutation of the LPS biosynthesis pathway, mutation of the K15 capsule pathway, mutation of the genes encoding for membrane proteins, but also the 22 different mutated genes), taking into account three observation conditions: *in vitro*, *in vivo* 10 h pi, and *in vivo* 48 h pi.

Analysis of variance (ANOVA) was employed to compare the maximum growth rates (MGRs) in the LB medium of the clones from three different conditions (*in vitro*, *in vivo* 10 h pi, and *in vivo* 48 h pi) and the WT strain 536, along with grazing scores. All statistical analyses were performed using GraphPad Prism software (v9.4.0) (GraphPad Software, USA), including Kaplan-Meier estimates of mouse survival. All individual animal data were included in the analysis. Survival differences were assessed using the Log-rank test, and significance was considered when the *P* value was <0.05.

## Data Availability

The raw data are available at https://doi.org/10.57745/LE4XNM, and the materials reported in this study are available upon request to the corresponding authors.

## SUPPLEMENTAL MATERIAL

Supplemental material jvi.01113-24-s0001.docx Supplemental figures and tables.

ASM does not own the copyrights to Supplemental Material that may be linked to, or accessed through, an article. The authors have granted ASM a non-exclusive, world-wide license to publish the Supplemental Material files. Please contact the corresponding author directly for reuse.
