# Supplementary material for: Variable fitness effects of bacteriophage resistance mutations in Escherichia coli: implications for phage therapy
Source: J Virol. 2024 Aug 30;98(10):e01113-24. doi: 10.1128/jvi.01113-24 (PMC11495123; doi:10.1128/jvi.01113-24)
Supplement: Supplemental material — Supplemental figures and tables. [file jvi.01113-24-s0001.docx]

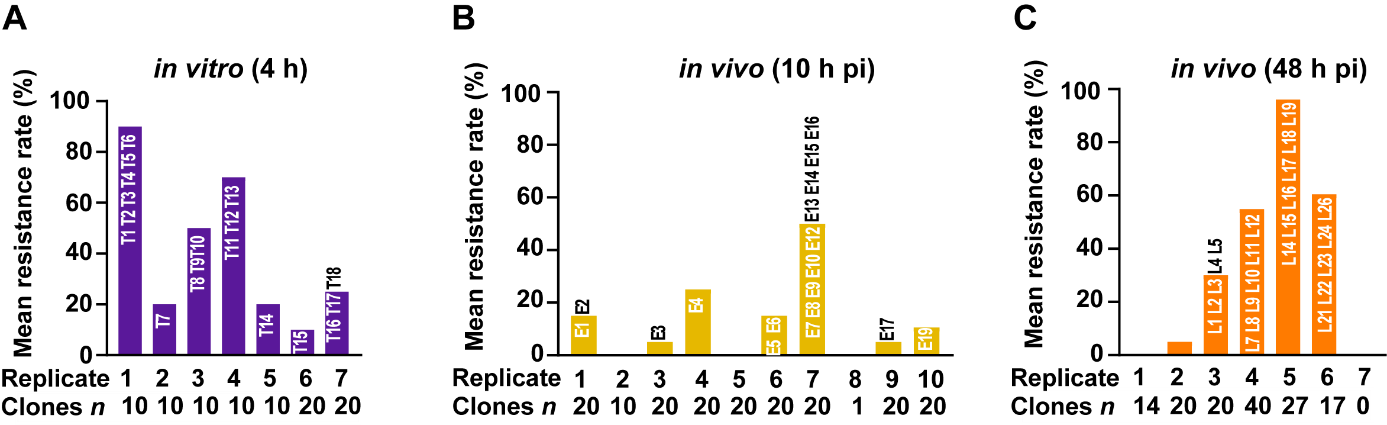


**Supplementary S1. Bacterial resistance rates to phage 536_P1 from the independent groups of both in vitro and in vivo conditions.**

**(A)** Seven liquid cultures of strain 536 were infected by phage 536_P1 at a phage:bacteria ratio of 1:10 for 4 h before sampling phage-resistant clones (n=10 to 20 per replicate). **(B, C)** A total of 17 BALB/cJRj mice were infected by a single dose of 1 x 10^8^ CFU of strain 536 and treated 2 h later by a single intranasal administration of phage 536_P1 (3 x 10^8^ PFU). Mice lungs were collected **(B)** 10 h (n=10) and **(C)** 48 h (n=7) pi to sample phage-resistant clones (up to 20 clones per mouse). The identity of sequenced clones (n=57) reported in this study and listed in **Supplementary S2** are indicated over the vertical bars. pi, post-infection.

| Clone^1^ | Mutation type | Nucleotide change | Gene | Protein change | SIFT | *In silico* prediction²  PROVEAN | POLYPHEN -2 | Function |
| --- | --- | --- | --- | --- | --- | --- | --- | --- |
| E16 | SNP | A→C | *ECP_0298* | Y160S | Tolerated (0.08) | Deleterious  (-6.782) | Probably damaging (1.000) | Surface adhesin precursor |
| E1 | SNP | T→C | *pqiA* | M314T | Affect protein function (0.04) | Deleterious  (-4.524) | Probably damaging (0.998) | Membrane stability |
| E9 | SNP | T→A | *mdtC* | L194Q | Affect protein function (0.00) | Deleterious  (-5.815) | Probably damaging (1.000) | Efflux system |
| L19 | SNP | A→G | *ygaH → / → mprA* |  |  |  |  | K15 capsule biosynthesis (transcriptional regulation) |
| L22 | indel | del 8494bp | *[ECP_3009]–[kpsE]* |  |  |  |  | K15 capsule biosynthesis (export) |
| L17 | SNP | G→T | *kpsD* | G32* |  |  |  | K15 capsule biosynthesis (export) |
| L2; L3; L4 | indel | del 1bp | *ECP_3027* |  |  |  |  | K15 capsule biosynthesis (export) |
| L24 | indel | del 353bp | *ECP_3035* |  |  |  |  | K15 capsule biosynthesis (export) |
| L19 | indel | del 1bp | *ECP_3029* |  |  |  |  | K15 capsule biosynthesis (synthesis) |
| T14; T10 | indel | del 1bp | *ECP_3031* |  |  |  |  | K15 capsule biosynthesis (synthesis) |
| E1 | indel | del 1bp | *ECP_3033* |  |  |  |  | K15 capsule biosynthesis (synthesis) |
| T15 | SNP | A→T | *ECP_3034* | W281R | - | Deleterious  (-7.875) | Probably damaging (1.000) | K15 capsule biosynthesis (synthesis) |
| E9 | SNP | T→G | *araA* | Q287P | Affect protein function (0.01) | Deleterious  (-5.997) | Probably damaging (0.998) | LPS biosynthesis |
| T18; L15 | SNP | C→T | *lpcA* | P78L | Affect protein function (0.00) | Deleterious  (-5.603) | Probably damaging (0.999) | LPS biosynthesis |
| L23 | indel | del 1bp | *galU* |  |  |  |  | LPS biosynthesis |
| E13 | indel | del 1bp | *galU* |  |  |  |  | LPS biosynthesis |
| T1 | indel | del 1bp | *galU* |  |  |  |  | LPS biosynthesis |
| E17 | indel | del 1bp | *galU* |  |  |  |  | LPS biosynthesis |
| T7; L11 | SNP | C→T | *galU* | Q274* |  |  |  | LPS biosynthesis |
| L10 | indel | del 1bp | *rfaE* |  |  |  |  | LPS biosynthesis |
| E19 | SNP | G→A | *rfaE* | T231I | Affect protein function (0.00) | Deleterious  (-5.917) | Probably damaging (1.000) | LPS biosynthesis |
| L9 | indel | del 1bp | *waaD* |  |  |  |  | LPS biosynthesis |
| T5 | SNP | G→A | *waaC* | W47* |  |  |  | LPS biosynthesis |
| E2 | indel | del 2468 bp | *waaV–waaT* |  |  |  |  | LPS biosynthesis |
| T17 | indel | del 1bp | *waaT* |  |  |  |  | LPS biosynthesis |
| E7 | SNP | C→T | *waaT* | W290* |  |  |  | LPS biosynthesis |
| E5 | SNP | T→C | *waaT* | D212G | Affect protein function (0.00) | Deleterious  (-6.863) | Probably damaging (1.000) | LPS biosynthesis |
| L24 | indel | del 1bp | *waaT* |  |  |  |  | LPS biosynthesis |
| L19 | indel | del 2bp | *waaT* |  |  |  |  | LPS biosynthesis |
| E9 | indel | del 1bp | *waaT* |  |  |  |  | LPS biosynthesis |
| T16; L12 | SNP | C→T | *waaT* | G177D | Affect protein function (0.00) | Deleterious  (-6.916) | Probably damaging (1.000) | LPS biosynthesis |
| T12 | SNP | C→A | *waaT* | E112* |  |  |  | LPS biosynthesis |
| L16 | SNP | G→T | *waaT* | Y105* |  |  |  | LPS biosynthesis |
| E16 | SNP | G→A | *waaT* | Q98* |  |  |  | LPS biosynthesis |
| T11; T13 | SNP | G→T | *waaT* | S80* |  |  |  | LPS biosynthesis |
| L26 | SNP | G→A | *waaT* | Q76* |  |  |  | LPS biosynthesis |
| L8 | indel | del 1bp | *waaT* |  |  |  |  | LPS biosynthesis |
| T8; T9 | SNP | C→T | *waaO* | W294* |  |  |  | LPS biosynthesis |
| L21 | SNP | C→T | *waaO* | W276* |  |  |  | LPS biosynthesis |
| T2; T4; T6 | SNP | G→T | *waaO* | S252* |  |  |  | LPS biosynthesis |
| E6; E10; E12 | indel | del 1bp | *waaO* |  |  |  |  | LPS biosynthesis |
| L5 | indel | del 1bp | *waaO* |  |  |  |  | LPS biosynthesis |
| L14 | indel | del 1bp | *waaO* |  |  |  |  | LPS biosynthesis |
| L18 | indel | +A | *waaO* |  |  |  |  | LPS biosynthesis |
| L7 | indel | del 54bp | *waaG* |  |  |  |  | LPS biosynthesis |
| E14; L1 | SNP | G→A | *waaG* | Q280* |  |  |  | LPS biosynthesis |
| L1 | SNP | G→A | *waaG* | R208C | Tolerated (0.06) | Deleterious  (-4.221) | Probably damaging (1.000) | LPS biosynthesis |
| E4 | indel | del 81 bp | *waaG* |  |  |  |  | LPS biosynthesis |
| E15 | indel | del 18bp | *waaG* |  |  |  |  | LPS biosynthesis |
| T3 | SNP | C→T | *waaG* | W71* |  |  |  | LPS biosynthesis |
| E3 | SNP | G→T | *waaG* | S58* |  |  |  | LPS biosynthesis |
| E8 | recombination |  | *galU* |  |  |  |  | LPS biosynthesis |
|  |  |  | *galU/hns* |  |  |  |  | LPS biosynthesis |
| E10 | recombination |  | *waaG* |  |  |  |  | LPS biosynthesis |
|  |  |  | *waaG* |  |  |  |  | LPS biosynthesis |

**Supplementary S2. List of the unique set of 53 unique mutations identified from the 57 phage-resistant clones sequenced.**

^1^ T#, clones from *in vitro* condition; E#, clones from *in vivo* (10 h pi); L#, clones from *in vivo* (48 h pi).

^2^ Predictions of the consequence of mutations on protein function.

| **Phage-resistant clones** | **Mutated genes** | **Predicted LPS structure** |
| --- | --- | --- |
| 536 WT | none | 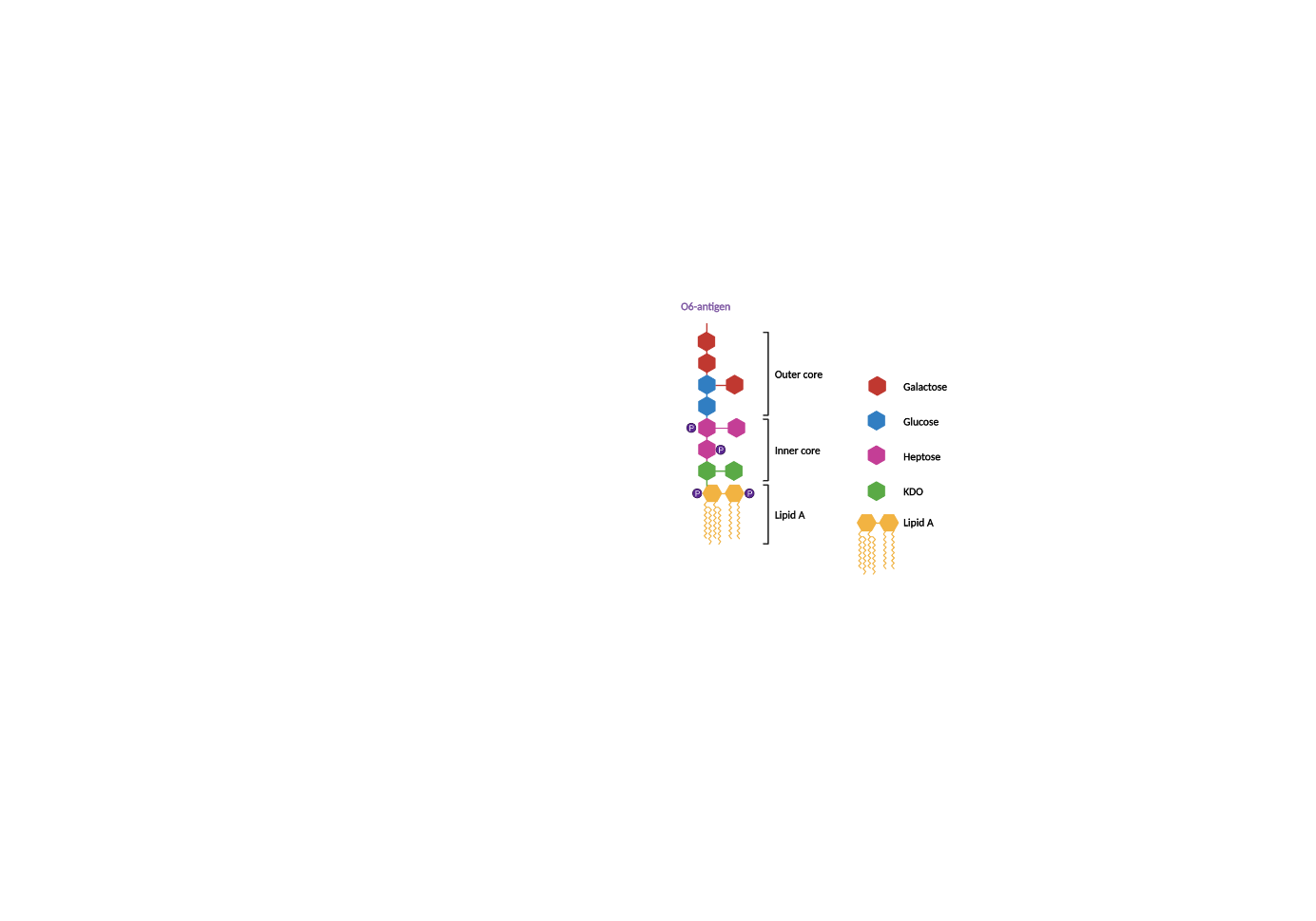 |
| T18. L15  E19. L10  L9 | *lpcA*  *rfaE*  *waaD* | 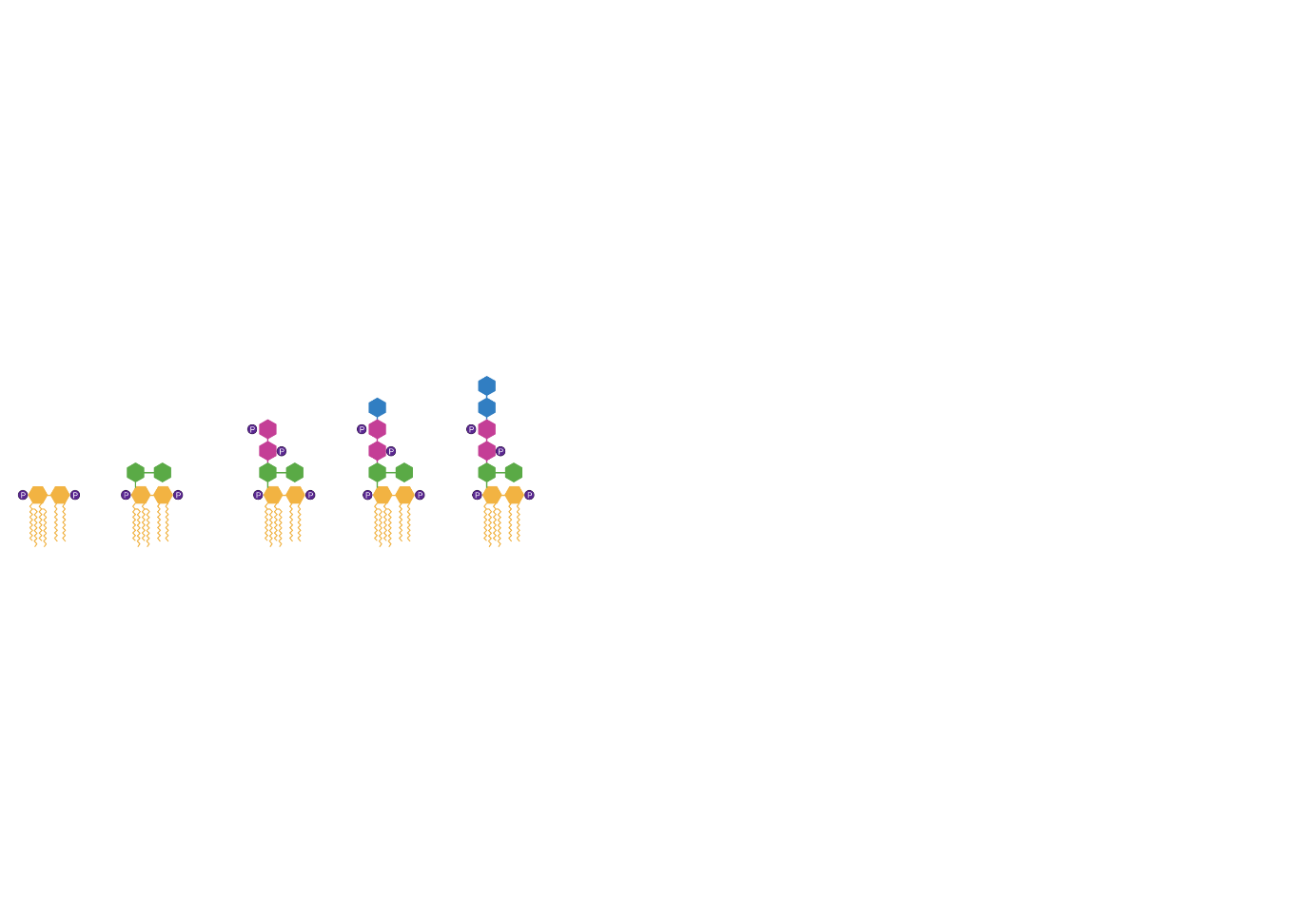 |
| T5 | *waaC* | 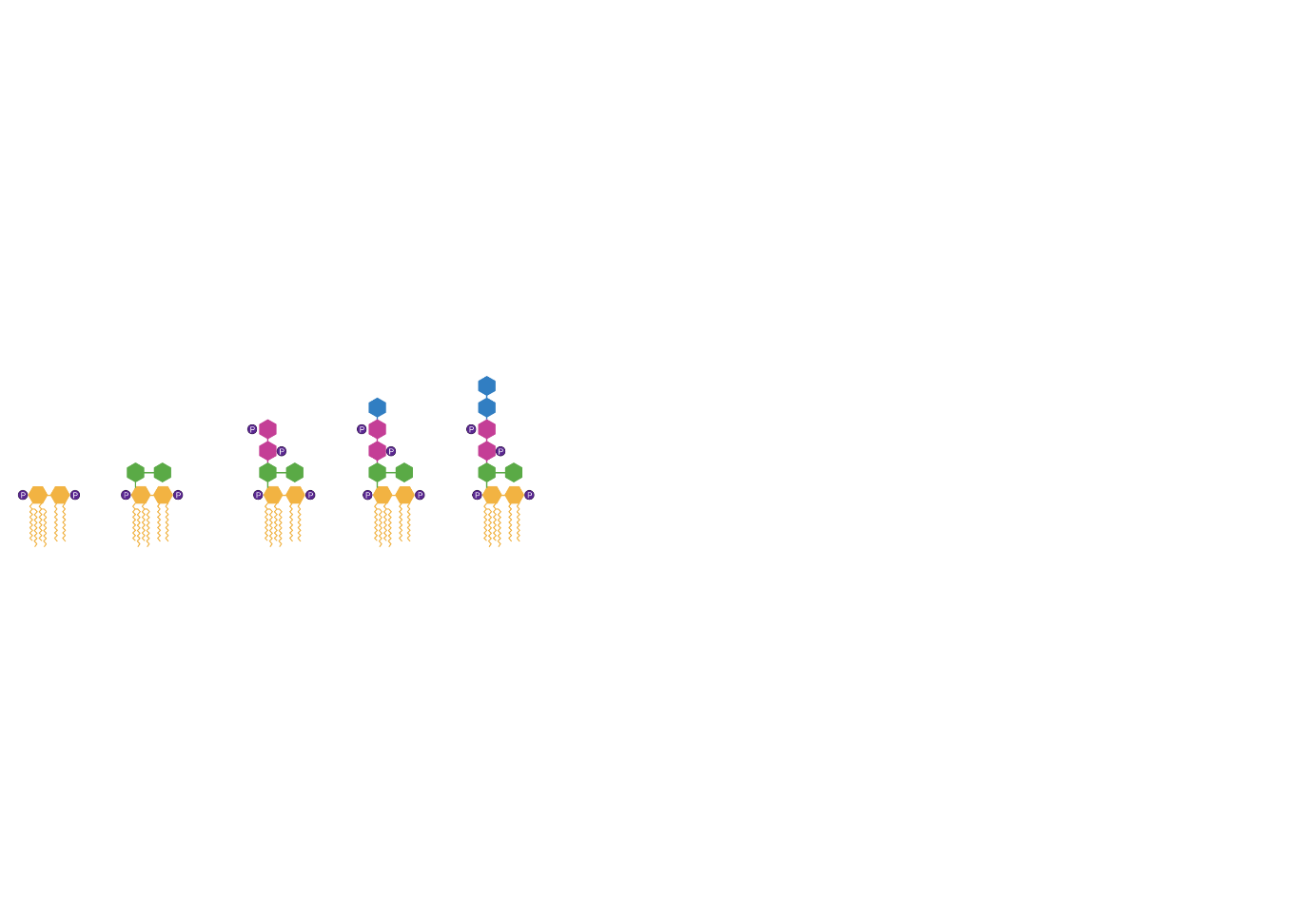 |
| T1. T7. E8. E13. E17. L11. L23  T3. E3. E4. E10. E14. E15. L1. L7 | *galU*  *waaG* | 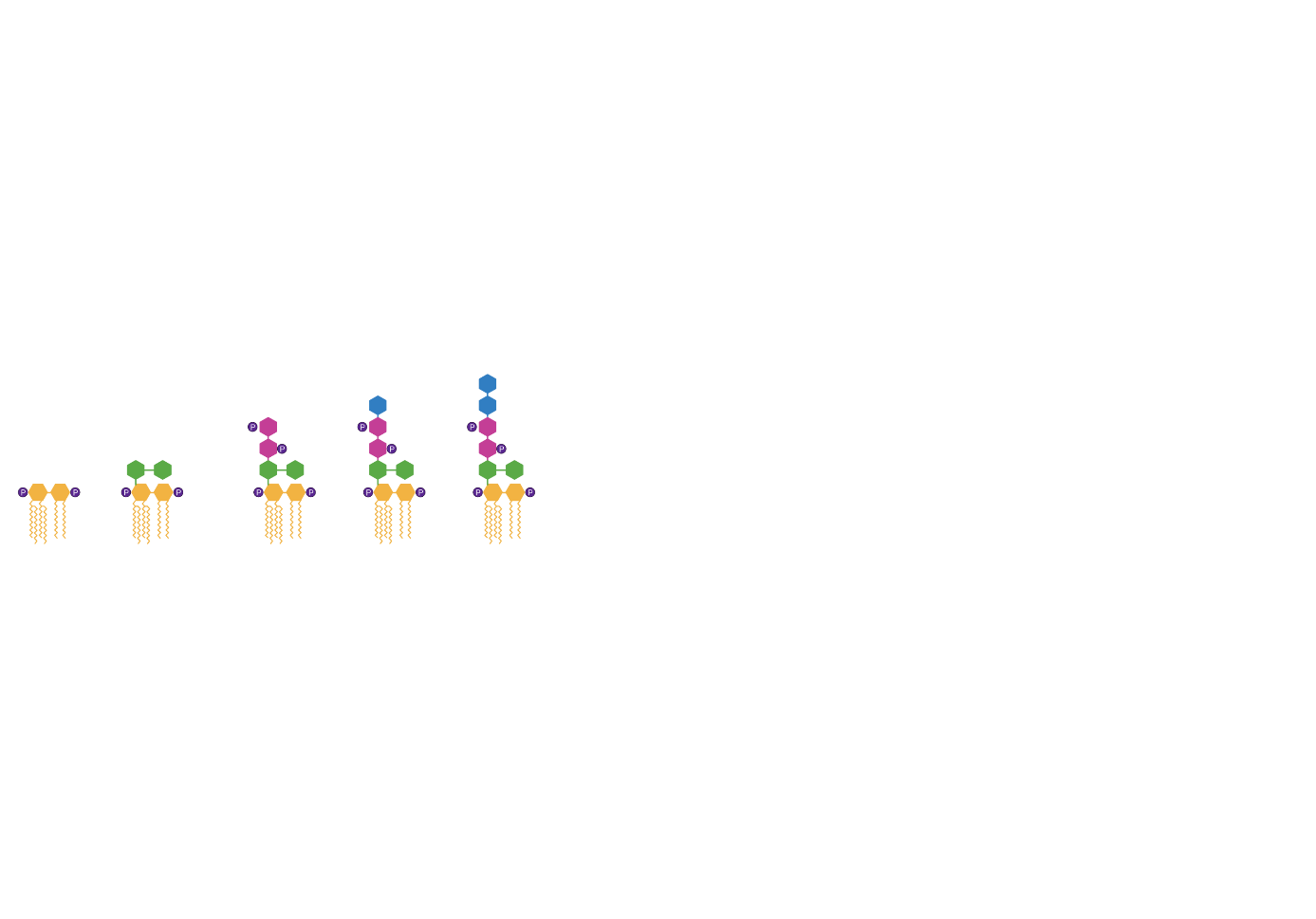 |
| T2. T4. T6. T8. T9. E6. E12. L5. L14. L18. L21 | *waaO* | 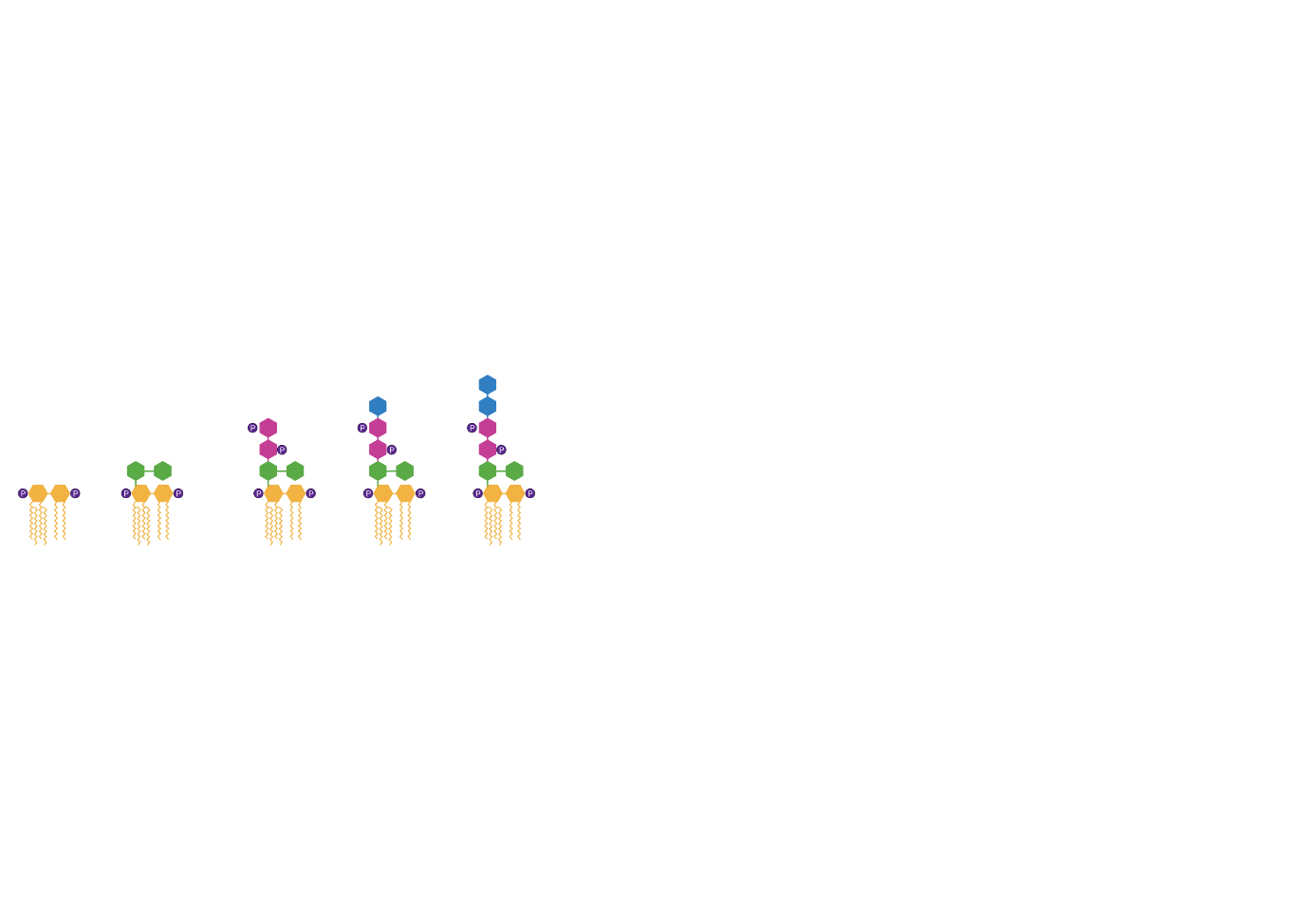 |
| T11. T12. T13. T16. E2. E5. E7. E9. E16. L8. L12. L16. L19. L24. L26 | *waaT* | 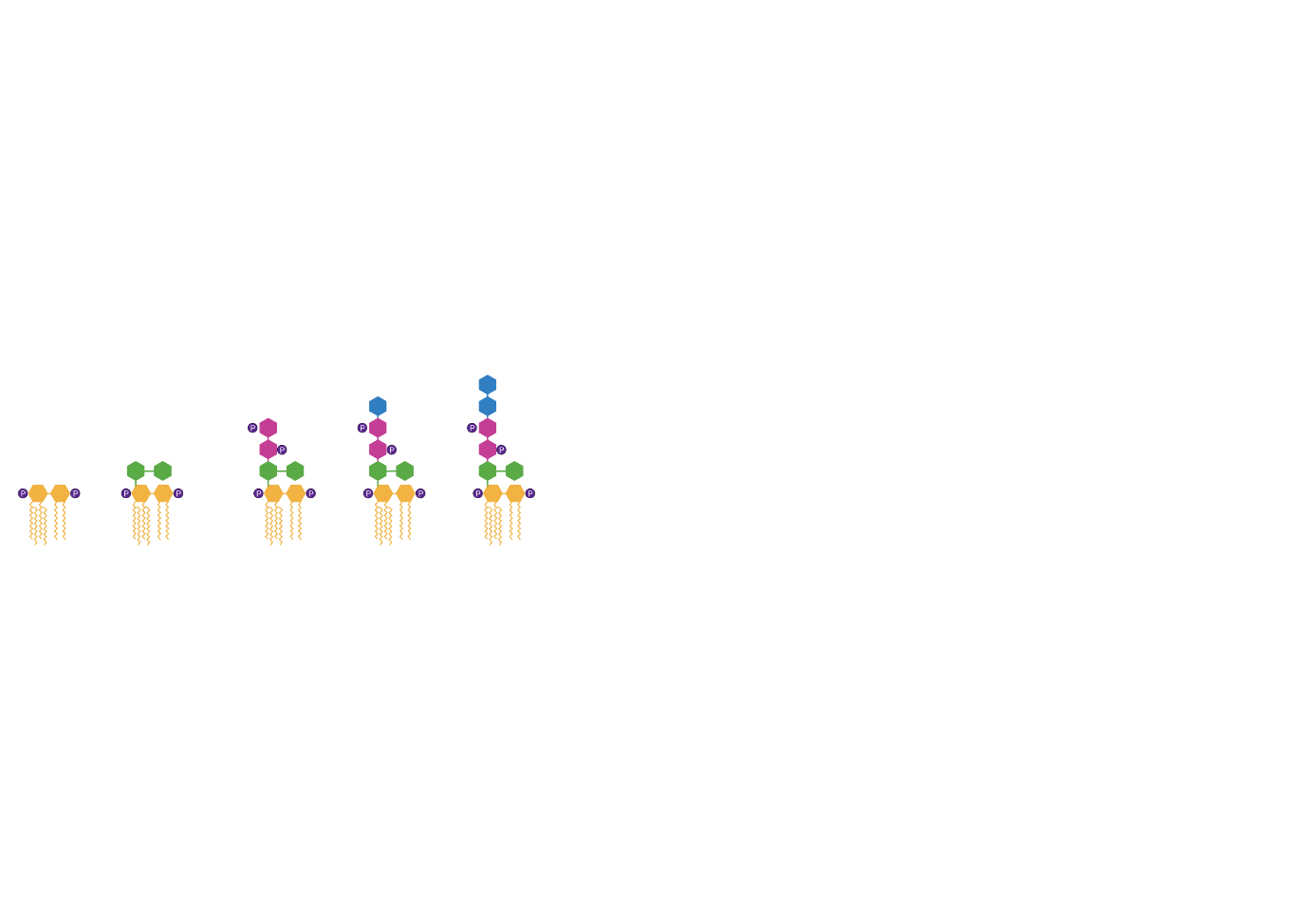 |

**Supplementary S3. The mutations of phage-resistant clones in the LPS biosynthetic genes are predicted to shorten the LPS structure.**

The table provides the list of 47 clones (left column) harboring a point mutation in genes related to the LPS biosynthesis pathway (middle column) and the predicted LPS structure (right column). LPS, lipopolysaccharide.


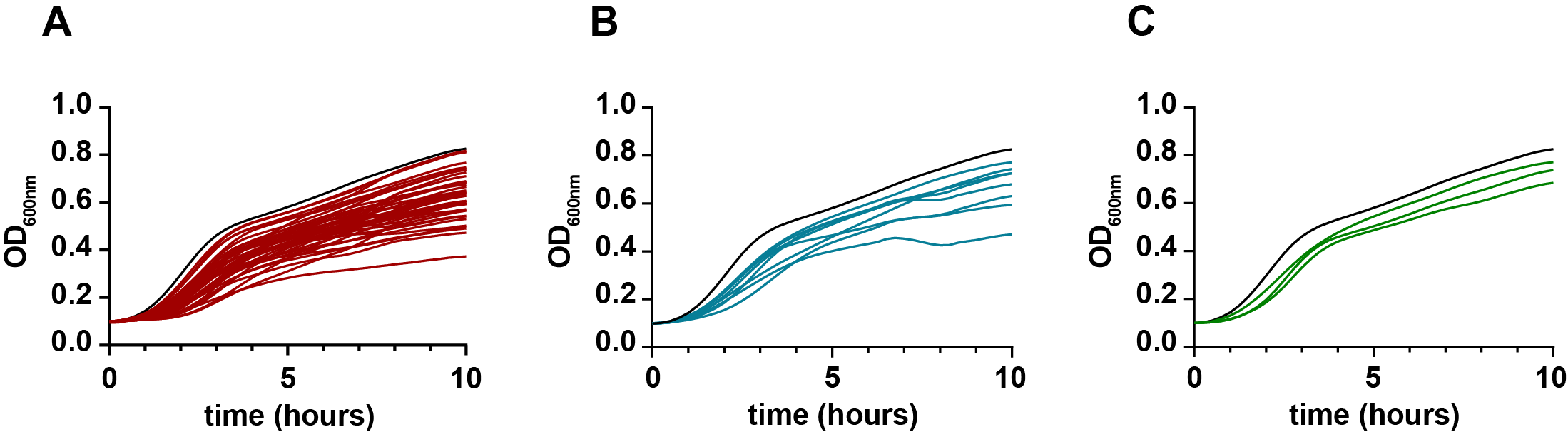


**Supplementary S4. The growth rate of phage-resistant mutants is decreased compared to the WT strain 536.**

The growth curves of all sequenced phage-resistant clones (*n*=47) with a unique set of mutations in a non-limited nutrient medium (LB medium) are displayed. The mean and smoothed growth curve of three independent replicates for each clone is compared to the WT strain 536 (black). Mutated functions cluster the representation: **(A)** LPS biosynthetic pathway (red), **(B)** K15 capsule locus genes (blue), and **(C)** membrane proteins (green). LPS, lipopolysaccharide.


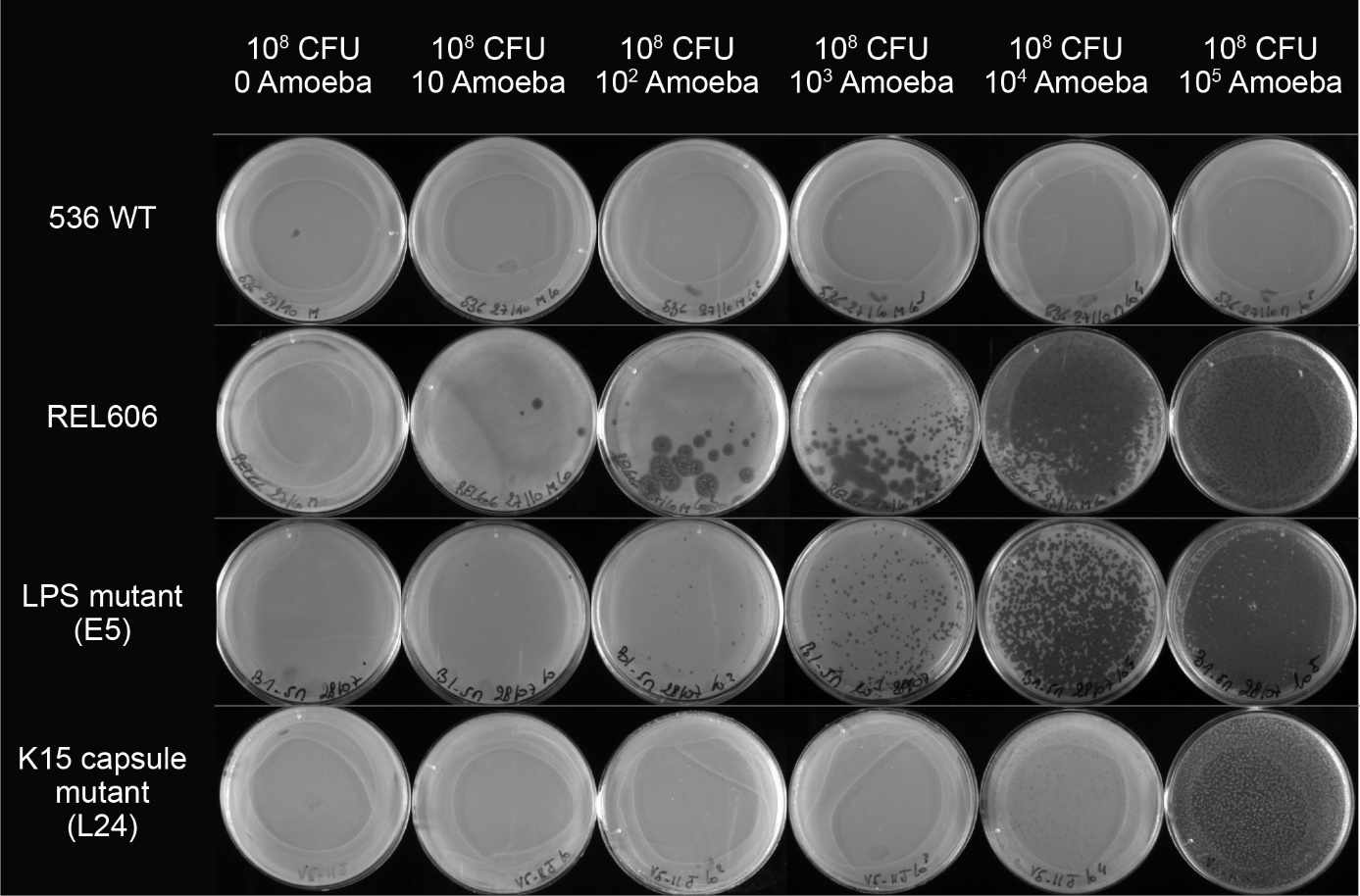


**Supplementary S5. Phage-resistant clones to 536_P1 become susceptible to protozoan predation by *Dictyostelium discoideum*.**

Representative images of grazing-resistant (strain 536) and grazing-susceptible (strain REL606) phenotypes are displayed (top two rows) together with two phage-resistant clones (E5 and L24) (bottom two rows). The serial dilution of amoeba cells ranges from 0 (left) to 1 x 10^5^ (right) on bacterial lawns (1 x 10^8^ CFU). LPS, lipopolysaccharide.

**
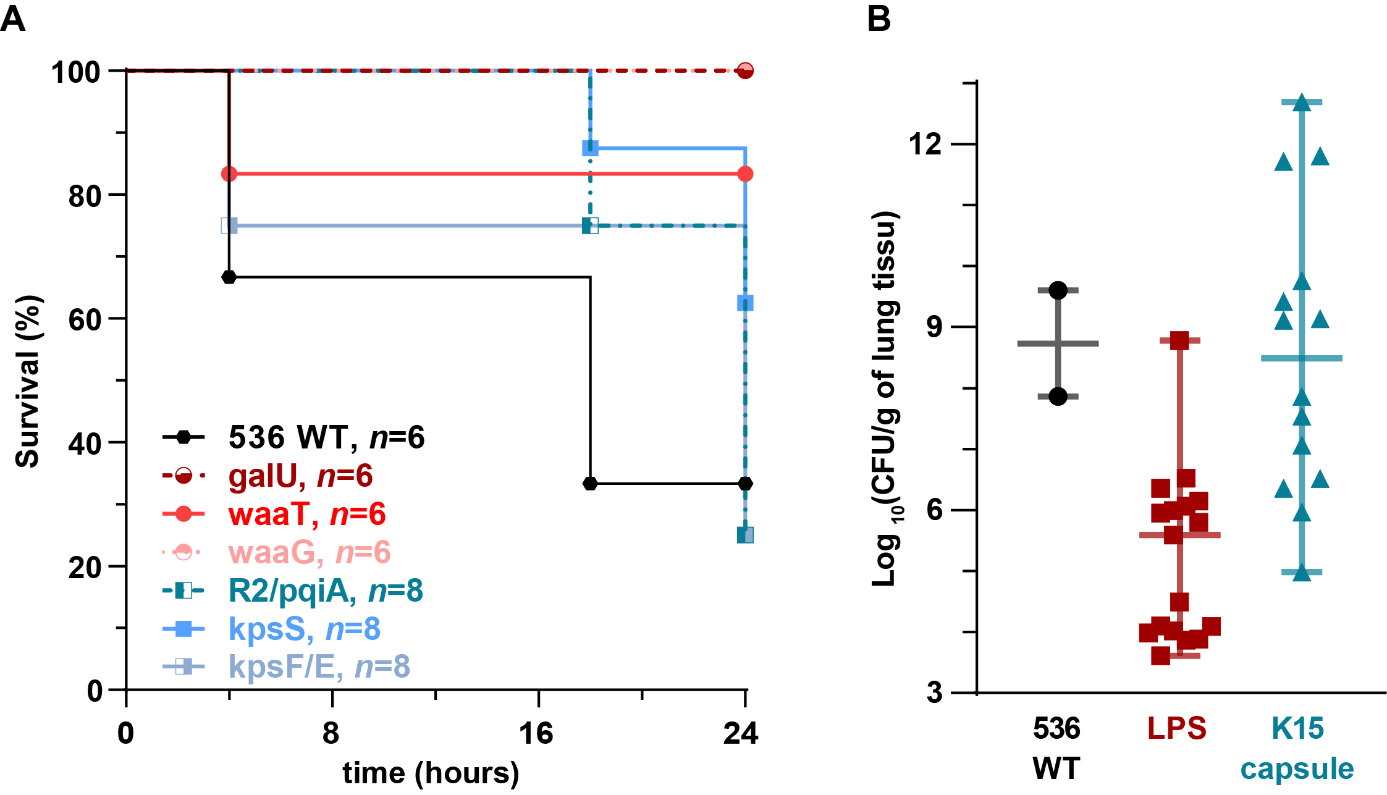
Supplementary S6. The virulence of LPS but not K15 capsule phage-resistant mutants is strongly attenuated.**

**(A)** To assess the virulence of phage-resistant clones, three LPS (*galU*, clone T1; *waaT*, clone T11; *waaG*, clone E15) and three K15 capsule (*ECP_3033*, clone E1; *ECP_3027*, clone L3; Δ(*ECP_3009–kpsE*), clone L22) mutants were used to infect BALB/cJRj mice with a single intranasal dose of 1 x 10^8^ CFU (*n*=6 to 8 per group), and survival rates was monitored up to 24 h pi. **(B)** Lungs from the infected mice described in (A) (*n*=2, 17, 14 for WT, LPS, and K15 clones, respectively) were collected at 24 h pi to count bacteria. LPS, lipopolysaccharide; pi, post-infection.

**Supplementary S7. List of the six phages used in this study.**

| **Bacteriophage** | **Morphology** | **Genus** | **Genome size (kb)** | **Genome accession number** |
| --- | --- | --- | --- | --- |
| 536_P1 | *Myoviridae* | *Phapecoctavirus* | 149.4 | OZ035728.1 |
| 536_P3 | *Podoviridae* | *Teetrevirus* | 39.7 | - |
| CLB_P2 | *Myoviridae* | *Dhakavirus* | 171.8 | OZ035781.1 |
| LF73_P1 | *Myoviridae* | *Tequatrovirus* | 168.9 | OZ035729.1 |
| LF110_P3 | *Myoviridae* | *Felixounavirus* | 87.1 | OZ035766.1 |
| DIJ07_P1 | *Myoviridae* | *Phapecoctavirus* | 142.8 | OZ035743.1 |


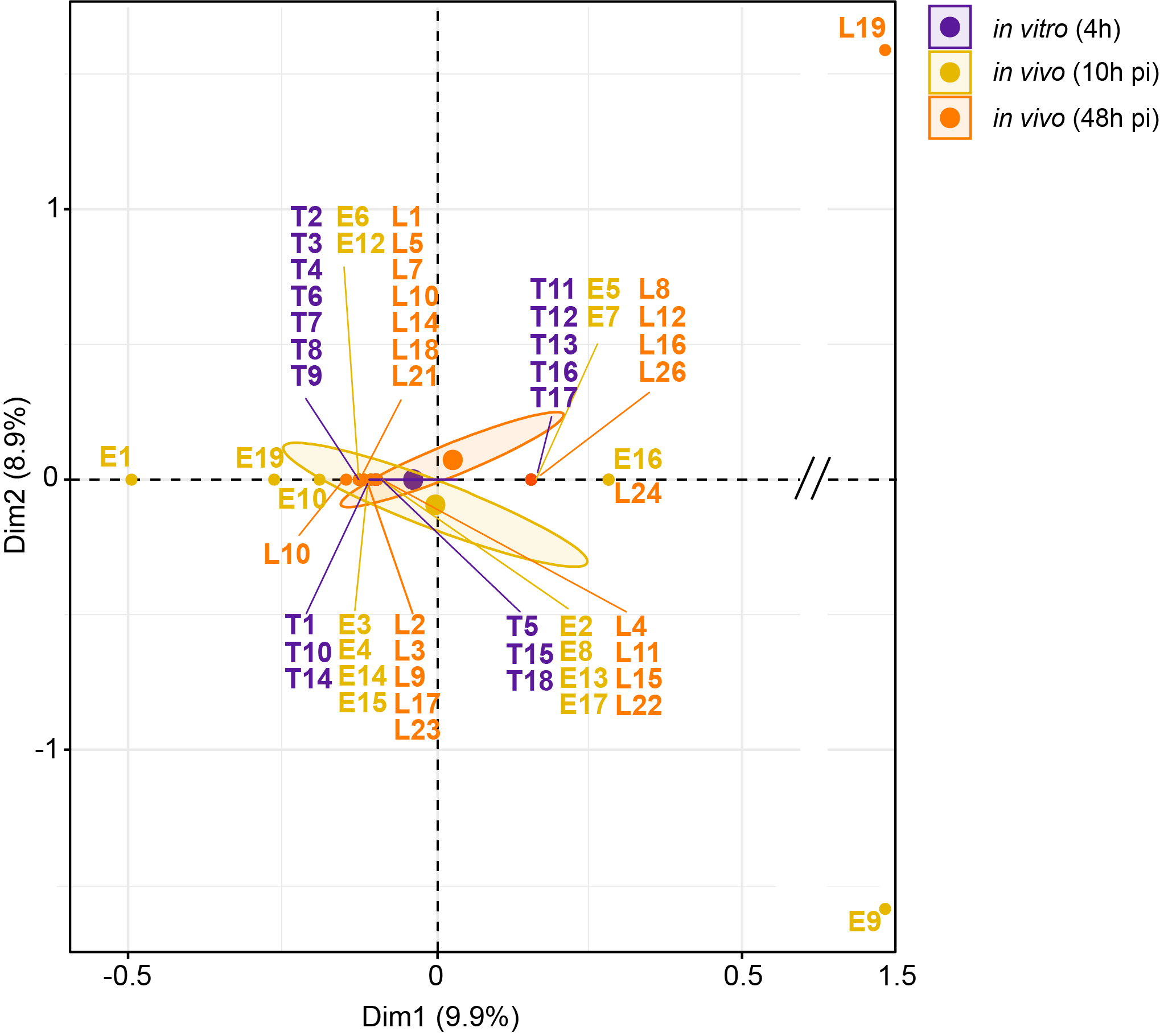


**Supplementary S8. Genome sequencing of the 57 phage-resistant clones reveals a mutational convergence of phage-resistance mechanisms at two levels (genes and biosynthetic pathways)**.

Multiple Correspondence Analysis (MCA) was conducted based on the mutated genes and biosynthetic pathways (LPS, K15 capsule, and membrane proteins) for each of the 57 clones. Mutated genes are color-coded according to conditions: *in vitro* (purple) and *in vivo* at 10 h (yellow) and 48 h pi (orange). LPS, lipopolysaccharide; pi, post-infection.


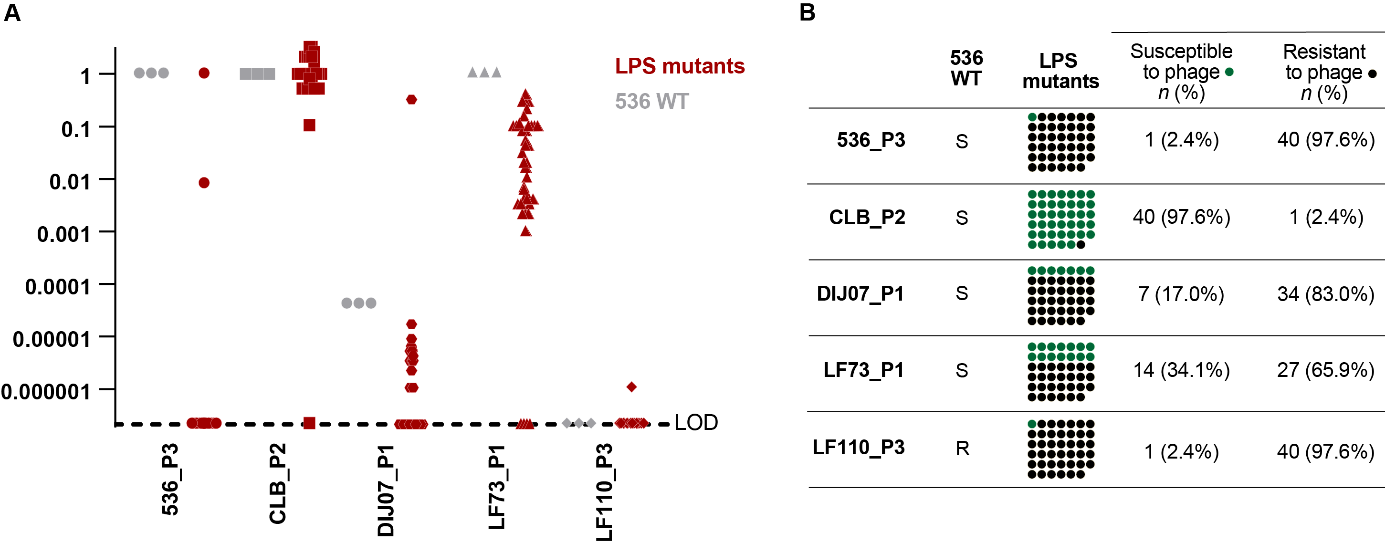


**Supplementary S9. Phage 536_P1 LPS-resistant clones display uneven susceptibility to five other phages.**

**(A)** Efficiency of plating (EOP) of five virulent phages (536_P3, CLB_P2, DIJ07_P1, LF73_P1, LF110_P3) on 41 phage 536_P1 resistant clones carrying a mutation in LPS biosynthetic genes, and three 536 WT clones. The EOP values correspond to the ratio of phage titers on the tested clone over the phage host strain. LOD, limit of detection of 10^-7^. **(B)** Synthetic representation of the EOP values from (A) with a cut-off value of 0.1 to differentiate susceptible (green) to resistant (black) phenotypes EOP, efficiency of plating; S, susceptible; R, resistant; LOD, limit of detection.
